# Likely Pathogenic/Pathogenic Variants in the Spliceosome Complex Genes SNRNP200, SF3B1, SF3B2, and SF3B4 Implicated in Nonsyndromic Orofacial Cleft

**DOI:** 10.1155/humu/2991452

**Published:** 2025-12-14

**Authors:** Peyman Ranji, Eleonore Pairet, Raphael Helaers, Pascal Brouillard, Bénédicte Bayet, Alexander Gerdom, Nicole Revencu, Miikka Vikkula

**Affiliations:** ^1^ Human Molecular Genetics, de Duve Institute, UCLouvain, Brussels, Belgium, deduveinstitute.be; ^2^ Centre Labio-Palatin, Division of Plastic Surgery, Cliniques universitaires Saint-Luc, UCLouvain, Brussels, Belgium, saintluc.be; ^3^ Center for Human Genetics, Cliniques universitaires Saint-Luc, UCLouvain, Brussels, Belgium, saintluc.be; ^4^ WELBIO Department, WEL Research Institute, Wavre, Belgium

**Keywords:** gene, mutation, nonsyndromic CLP, *SF3B1*, *SF3B2*, *SF3B4*, *SNRNP200*, spliceosome

## Abstract

The genetic basis of nonsyndromic orofacial cleft (NsOFC) remains elusive, although associations have been identified with various genetic loci. NsOFC has a less pronounced genetic background than syndromic orofacial cleft (SyOFC), albeit Mendelian inheritance has been identified. Our hypothesis was that genes related to spliceosome function may contribute to NsOFC pathophysiology, as they do for some syndromic cases. Exome sequencing was conducted on 224 unrelated NsOFC probands. We performed filtering and analyses of predicted pathogenicity of rare variants using Highlander. We focused on 26 genes encoding spliceosome proteins. Subsequently, bioinformatic tools, such as AlphaFold, and PyMol, were applied to generate three‐dimensional structures to interpret the effects of the identified variants on protein structure and interaction domains. We found six likely damaging variants: three heterozygous missense variants in small nuclear ribonucleoprotein U5 200 kDa subunit (*SNRNP200*) in three multiplex NsOFC families, and two missense and one splice site variant in splicing factor 3b subunit 1 (*SF3B1*), 4 (*SF3B4*), and 2 (*SF3B2*) in two posterior CP families and a complete CP patient, respectively. These results suggest that variants in the spliceosome complex genes, observed in 2.7% of NsOFC cases in our cohort, may contribute to disease susceptibility as potential risk factors.

## 1. Introduction

Orofacial cleft (OFC), which includes cleft lip with or without cleft palate (CL/P) and cleft palate only (CP), is the most common craniofacial birth defect, with an incidence of about 1/700 live births. OFC is generally divided into two groups: syndromic OFC (SyOFC) and nonsyndromic OFC (NsOFC), depending on whether the OFC is associated with other anomalies or not [1]. SyOFC often follows Mendelian inheritance, while NsOFC is thought to have a complex multifactorial etiology [1]. Next‐generation sequencing technologies, such as exome sequencing (ES), have allowed substantial progress in the identification of genetic variants associated with SyOFC and NsOFC.

Prior to ES, several genome‐wide association studies (GWAS) identified more than 40 loci implicated in nonsyndromic cleft lip with or without cleft palate (NsCL/P). These loci account for around 30% of the heritability of NsOFC [2–4]. An important part of the missing heritability could be due to rare variants in NsCL/P patients not detected by the GWAS approach. However, the high locus heterogeneity and reduced penetrance hinder studies that aim to associate pathogenic variants with NsOFC. In contrast, in SyOFC, genetic factors often play a strong Mendelian role, emphasizing the importance of continued efforts to identify novel genes. These become prime candidates to study in NsOFC. Using ES in cohort studies, causative pathogenic variants have been identified in about 10% of NsOFC subjects in genes known to cause SyOFC [5, 6].

Genes involved in spliceosome function and related aspects of mRNA processing have been implicated in some SyOFCs [7–10], but not yet in NsOFC. For instance, splicing factor 3b subunit 2 (*SF3B2*) and splicing factor 3b subunit 4 (*SF3B4*) are associated with craniofacial microsomia (MIM:164210; SyCL/P), and Nager syndrome (MIM:154400; SyOFC), respectively [11]. Additional spliceosomal components linked to SyOFC include small nuclear ribonucleoprotein polypeptide E (*SNRPE*; associated with mandibulofacial dysostosis with microcephaly, MIM: 618733), small nuclear ribonucleoprotein polypeptide B (*SNRPB;* cerebro–costo–mandibular syndrome, MIM: 117650), Poly(U) Binding Splicing Factor 60 (*PUF60;* Verheij syndrome, MIM: 613976), and zinc finger CCCH‐Type, RNA binding motif and serine/arginine rich 2 (*ZRSR2;* variants reported in syndromic presentations with craniofacial features) [11, 12]. Small nuclear ribonucleoprotein U5 200kDa subunit (*SNRNP200*), on the other hand, has been linked to retinitis pigmentosa (RP, MIM: 610359), which does not feature craniofacial anomalies [11]. Notably, mutations in some spliceosome‐related genes, such as *CWC27* (retinitis pigmentosa with craniofacial anomalies, MIM: 617073), OFD1 (orofaciodigital syndrome I, MIM: 311200), and *RNU4ATAC* (Taybi–Linder syndrome, MIM: 210710; Roifman syndrome, MIM: 616541), demonstrate overlapping roles in different tissues, leading to both retinal degeneration and craniofacial abnormalities, including orofacial clefts [12, 13]. This overlapping functionality underscores the need to investigate whether such genes also contribute to the etiology of NsOFCs. To address this, we searched for rare variants predicted to be pathogenic through bioinformatic analyses in a cohort of 224 unexplained NsOFC cases. We modeled the identified variants in silico to predict their effects on 3D protein structure and performed co‐segregation studies to estimate penetrance.

## 2. Methods

### 2.1. Sample Selection

Clinical data and blood DNA samples from NsOFC patients and their family members were collected at the Centre Labio‐Palatin, Cliniques Universitaires St Luc, Brussels, Belgium, and Amiens‐Picardie Hospital, France. Participants signed an informed consent approved by institutional review boards. Each patient completed a standardized questionnaire, and clinician(s) evaluated family history and clinical phenotype. The ethical committee of the medical faculty at the University of Louvain approved the research protocol. The study included patients who underwent genetic testing (karyotype and/or 22q deletion [MIM:188400] testing, and/or interferon regulatory factor 6 [*IRF6*] or grainyhead like transcription factor 3 [*GRHL3*] sequencing) and those without prior genetic testing. ES was performed on 335 DNA blood samples including 224 probands and 111 affected NsOFC member(s) and/or unaffected member(s) from 224 different families (97 familial cases and 127 sporadic). Additional blood samples were collected for co‐segregation studies for families with an identified putative pathogenic variant in spliceosome genes.

### 2.2. Exome Sequencing

DNA isolation, DNA quantification, ES details, and Fastq file analysis were carried out following the established protocols outlined in previous publications [14, 15]. The data was uploaded to Highlander (http://sites.uclouvain.be/highlander/) to perform detailed filtering of variants.

### 2.3. Variant Filtering

For 335 samples from 224 different NsOFC families, filtering was performed using Highlander. Genes in the spliceosome complex were selected as candidate genes for NsOFC on the basis of three criteria: (i) reported in review articles as related to or mutated in craniofacial disorders, (ii) identified through close protein–protein interactions with known CL/P‐related genes or pathways (BioGRID), or (iii) protein‐coding genes with strong constraints (gnomAD v4.1.1: pLI ≥ 0.9 and/or missense Z ≥ 3.1). (Supporting Table S1) [7, 11, 12, 16–19]. In addition to the primary 24‐gene panel thus selected, we examined two additional spliceosomal genes (*ZRSR2* and *SNRPE*), which were recently implicated in craniofacial spliceosomopathies [20, 21]. Variants were filtered using GATK’s standard quality‐control criteria, retaining only those marked “PASS.” Filters included thresholds for Quality by Depth (QD ≥ 2.0), Fisher Strand Bias (FS ≤ 60.0), and Mapping Quality (MQ ≥ 40.0) to ensure high‐confidence calls. Filtering for possible pathogenic variants was performed using an allele frequency of < 1% in the Genome Aggregation Database (gnomAD v4.1.0) (https://gnomad.broadinstitute.org), Regeneron Genetics Center (RGC) (https://rgc-mcps.regeneron.com/), and deCODE Allele Frequency (deCAF) (https://decaf.decode.com/).

Filtering utilized an internal database of 3819 individuals with 51 different non‐cleft pathologies, excluding variants present in more than three pathologies. Missense variants were considered deleterious if predicted as such by at least 8 out of 20 algorithms in Highlander (DAMAGING in Mutation Taster, FATHMM, FATHMM‐XF, Polyphen2 (HDIV), Provean, SIFT4G, Mutation Assessor, MCAP, LRT, Lists2, Deogen, ClinPred, BayesDel (with MaxMAF), PrimateAl and MetaSVM, or a score > 20 in CADD Phred, > 0.5 in VEST, > 0.5 in REVEL, > 0.75 in MVP, > 0.75 in MutPred), computed as consensus prediction (Supporting Table S2, S3 and Supporting Figure S1).

### 2.4. Segregation Studies

Segregation analysis of the identified variants was performed on the affected and unaffected family members by ES or Sanger sequencing on an ABI 3130XL genetic analyzer (Applied BioSystems).

### 2.5. In Silico Structural Modeling and Prediction

AlphaFold v2.3.2 was applied to generate three‐dimensional structures of the proteins with missense mutations [22]. The protein 3D models containing wild‐type and missense variants were analyzed in PyMOL. The top mutant model was chosen based on alignment [23]. HOPE web service was used for analyses of structural effects of missense variants in proteins [24]. In silico analyses using mCSM‐Stability, DynaMut2, and DDMut assessed protein stability changes (ΔΔG) for each variant. Additionally, mCSM‐PPI and mmCSM‐NA were applied for protein–protein and protein–RNA interaction affinities (Supporting Tables S4 and S5) [25–28]. ΔΔG is a thermodynamic measure indicating the change in protein stability (Gibbs free energy) when mutated, with zero as cutoff (*Δ* *Δ*G < 0: destabilizing; *Δ* *Δ*G > 0: stabilizing). The range of ΔΔG varies depending on the specific mutation and its impact on protein structure [25–28]. Arpeggio was employed for molecular interaction analysis (Supporting Table S6), offering comprehensive insights into variant impacts on protein structure and function [29].

### 2.6. ACMG Categorization

The American College of Medical Genetics and Genomics/Association for Molecular Pathology (ACMG/AMP) guidelines, comprising 28 criteria, were applied to assess and interpret variant pathogenicity. Each variant was evaluated using a combination of evidence levels: pathogenic (P), likely pathogenic (LP), variant of uncertain significance (VUS), likely benign (LB), and benign (B). Criteria were categorized as supporting, moderate, or strong based on the strength of evidence. For instance, PM1 (moderate) was applied to variants located in critical functional domains or mutational hotspots, PM2 (moderate) was used for variants absent or extremely rare in population databases (e.g., gnomAD), and PP2 (supporting) was applied to missense variants in genes with a low rate of benign missense variation and where missense changes are a known mechanism of disease. Computational predictions supported the application of PP3 (moderate) when multiple tools consistently indicated a deleterious effect. Variants were classified as likely pathogenic if they met the threshold of 3 moderate criteria, 1 strong + 1 moderate, 1 moderate + 4 supporting, or 2 moderate + 2 supporting criteria [30].

## 3. Results

**Figure 1:**
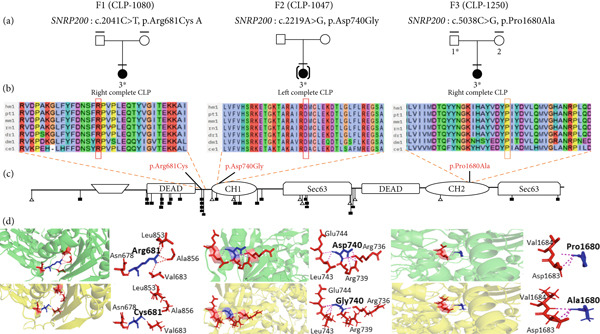
The genealogical trees and alignment of SNRNP200 orthologous amino acid sequences of various species around the identified Arg681Cys, Asp740Gly, and Pro1680Ala substitutions and three‐dimensional modeling of wild‐type and mutant domains. (a) Family number; mutated gene: variant; marked above pedigrees. Clinically studied individuals, horizontal bar; available blood sample, numbered individual; ∗, mutation carrier, [ ], adopted index. (b) Arg681, Asp740, and Pro1680 highly conserved in different species. (c) SNRNP200 protein domains. Retinitis Pigmentosa variants below. Type of mutation and pathologic effect, by shape, filled symbol, likely pathogenic/pathogenic; empty symbol, variant of unknown significance; triangle, frameshift or nonsense variant; square, missense variant. (d) Green, wild‐type 3D structure; yellow, mutant 3D structure. Hydrogen‐binding contacts (pink dashed lines); wild‐type and mutant amino acid at position 681, 740, and 1680 (blue), all interacting amino acids (red), labeled. Mutant model, Cys681 detached from interacting amino acids Val683, Gly854, and Ala856 compared to wild‐type model. Mutant model, Gly hydrogen bond detached from interacting amino acids. Mutant model, Ala attached to two amino acids (Val684 and Asp1683), while Pro only binds to Asp1683.

**Figure 2:**
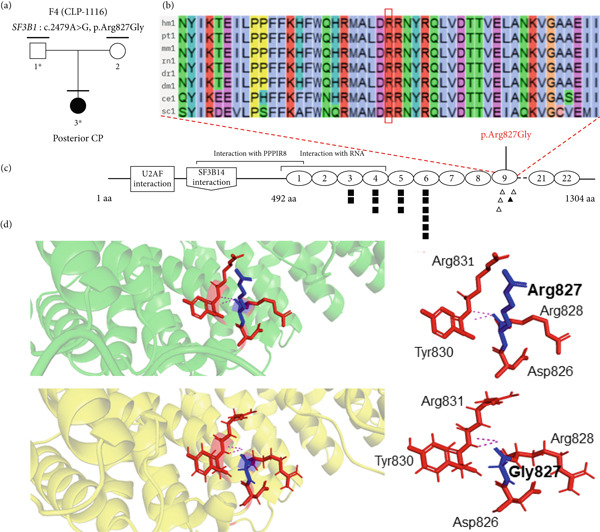
The genealogical tree and alignment of SF3B1 orthologous amino acid sequences of various species around the identified Arg827Gly substitution and three‐dimensional modeling of wild‐type and mutant domains. (a) Family number; mutated gene: variant; marked above pedigrees. Clinically studied individuals, horizontal bar; available blood sample, numbered individual; ∗, mutation carrier. Cleft palate (CP). (b) Arg827 highly conserved in different species. (c) SF3B1 protein domains and cancer variants in SF3B1. Filled symbol, likely pathogenic/pathogenic; empty symbol, variant of unknown significant; triangle, frameshift or nonsense variant in chronic lymphocytic leukemia; square, missense variant. Arg. (d) Green, wild‐type 3D structure; yellow, mutant 3D structure. Hydrogen‐binding contacts (pink dashed lines) of Arg827 (blue) and all interacting amino acids (red) labeled. Mutant and wild‐type model, both mutant and wild‐type attached to similar amino acids at a different pattern.

**Figure 3:**
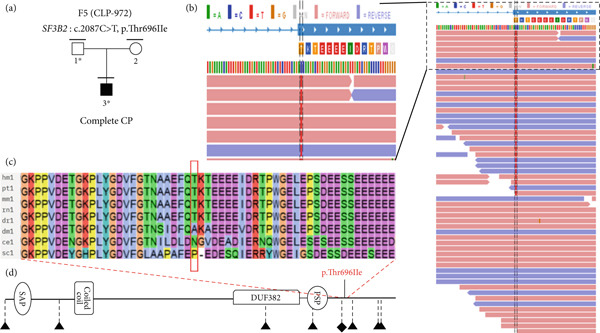
The genealogical trees and alignment of SF3B2 orthologous amino acid sequences of various species around the identified Thr696 substitution. (a) Family number; mutated gene: variant; marked above pedigrees. Clinically studied individuals, horizontal bar; available blood sample, numbered individual; ∗, mutation carrier. (b) Alignment of variant present in forward and reverse reads (red and blue, respectively). (c) Thr696 conserved in four species. (d) SF3B2 protein domains and variants; p.Thr696Ile variants and different diseases in protein domains; triangle, loss‐of‐function and likely pathogenic variants in craniofacial microsomia; diamond, loss‐of‐function and likely pathogenic variants in myelodysplastic syndrome.

**Figure 4:**
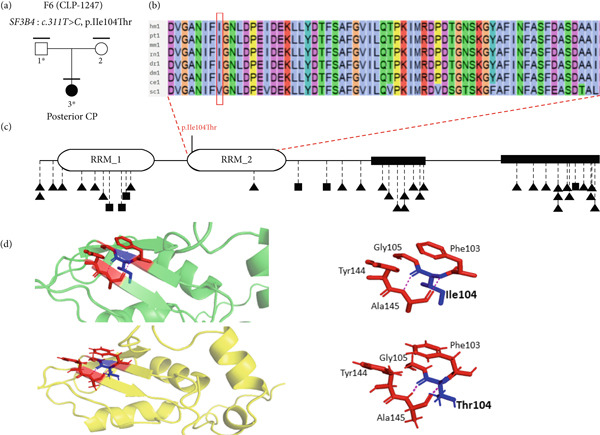
The genealogical trees and alignment of SF3B4 orthologous amino acid sequences of various species around the identified p.Ile104Thr substitution. (a) Family number; mutated gene: variant; marked above pedigrees. Clinically studied individuals, horizontal bar; available blood sample, numbered individual; ∗, mutation carrier. (b) Ile104 conserved in three species. (c) SF3B4 protein domains and variants; Ile104Thr of SF3B4 and likely pathogenic/pathogenic variants in Nager syndrome. Triangle: frameshift, nonsense or splice site variant; square: missense variant. (d) Green, wild‐type 3D structure; yellow, mutant 3D structure. Hydrogen‐binding contacts (pink dashed lines); wild‐type and mutant amino acid at position 104 (blue), all interacting amino acids (red) are labeled. Mutant model, Ile104 and Thr104 binds to Ala145 in the same pattern.

Out of the 224 unrelated families with NsOFC, 24 families were solved using a list of 554 well‐known candidate genes (Supporting Table S7). Of these, 20 families were solved by identifying variants in SyOFC genes (unpublished data), and four families were solved by identifying putative pathogenic variants in *ARHGAP29* [15]. In addition, we found six different rare heterozygous missense variants with evidence for pathogenicity in the 26 spliceosomal candidate genes (Table 1 and Supporting File S8). Three of them were identified in the *SNRNP200* gene in three unrelated NsCLP patients. *SNRNP200* (HGNC: 30859) is intolerant to LoF, evidenced by a pLI value of 1.00, and highly sensitive to missense variants with Z‐score of 8.77 in gnomAD (Figure 1). Another two missense variants were identified in *SF3B1* (HGNC: 10768) and *SF3B4* (HGNC:10771) in a Pierre Robin sequence (PRS) and a posterior CP family, respectively, and a nucleotide substitution in an exonic splice site region in *SF3B2* (HGNC:10769) in a complete CP patient (Figure 2, Figure 3 and Figure 4). The Z‐score for missense variants in *SF3B1*, *SF3B2*, and *SF3B4* are 9.84, 4.57, and 4.19, respectively, and pLI values for all are 1.00.

**Table 1:** Characteristics of genetic variants identified in index subjects and parents.

**Gene**	**Sample**	**Phenotype**	**Hgvs_dna**	**Hgvs_protein**	**Consensus_prediction**	**Exon number**	**Father**		**Mother**		**Allele count**				**ACMG**
							**Carrier (+/−)**	**Phenotype**	**Carrier (+/−)**	**Phenotype**	**gnomAD**	**RGC** ^**a**^	**deCAF** ^**b**^	**Local DB** ^**c**^	
SNRNP200^**d**^	CLP‐1080‐3	Complete Right CLP	c.2041C>T	p.Arg681Cys	20	16/45	N.A	Normal	N.A	Normal	1	1	0	0	P
	CLP‐1047‐3	Complete Left CLP	c.2219A>G	p.Asp740Gly	19	17/45	N.A	Normal	N.A	Normal	0	0	0	0	LP
	CLP‐1250‐3	Complete Right CLP	c.5038C>G	p.Pro1680Ala	17	36/45	+	Normal	—	Normal	5	10	0	0	LP
SF3B1^**e**^	CLP‐1116‐3	Posterior CP (PRS)	c.2479A>G	p.Arg827Gly	18	17/25	+	Normal	—	Normal	0	0	0	0	LP
SF3B2^**f**^	CLP‐972‐3	Complete CP	c.2087C>T	p.Thr696Ile	5	18/22	—	Normal	+	Normal	7	13	0	1	LP
SF3B4^**g**^	CLP‐1247‐3	Posterior CP	c.311T>C	p.Ile104Thr	16	3/6	+	Normal	—	Normal	14	17	0	0	LP

Abbreviations: CP, cleft palate; CLP, cleft lip and palate; PRS, Pierre Robin sequence; N.A, not available; P, pathogenic; LP, likely pathogenic.

^a^RGC, Regeneron Genetics Center.

^b^deCAF: deCODE allele frequency.

^c^DB: database.

^d^NM_014014.5.

^e^NM_012433.4.

^f^NM_006842.3.

^g^NM_005850.5.

### 3.1. SNRNP200

#### 3.1.1. Family 1

The proband (CLP‐1080‐3) had a right complete cleft lip and palate. The parents’ clinical examination was unremarkable, and there was no family history of NsOFC. At the age of 11 years, the index patient had no sign of RP and no deletion in 10p14 or 22q13. The index person had a heterozygous nucleotide change (NM_014014.5: *c.2041C>T*) in exon 16 of *SNRNP200*. DNA from the parents was not available for testing (Figure 1a).

The variant is predicted to code for a p.(Arg681Cys) with the maximal consensus score for pathogenicity of 20 out of 20 algorithms, i.e., all in silico algorithms predicted this variant to be damaging (Supporting Table S2). This variant was present only once among the 628,768 alleles in gnomAD and the 1,683,926 alleles in the RGC database (alternative allele frequency [AAF] < 0.00001). Moreover, this variant was not found in deCAF or among the 3 819 samples of the 51 noncleft pathologies in the local Highlander database (Table 1). The variant is reported nine times in ClinVar (https://www.ncbi.nlm.nih.gov/clinvar/) for RP (5 times as pathogenic and 4 times as likely pathologic), 58 times in LOVD for RP (31 times pathologic and 26 times likely pathologic), and also once in Geno2MP (http://geno2mp.gs.washington.edu) for RP [31].

The Arg681 is located in a highly conserved position of SNRNP200 across different species (Figure 1b). The mutant residue (cysteine) is smaller and more hydrophobic than the wild‐type residue (arginine). Moreover, the wild‐type residue is positively charged, while the mutant residue is neutral. The variant is located in the ATP binding domain of the C‐terminal helicase 1 (CH1) (Figure 1c). The p.(Arg681Cys) substitution leads to reduced interactions, including fewer van der Waals (VdW) clashes, hydrogen bonds and hydrophobic interactions, as determined by Arpeggio. (Supporting Table S6). In the wild type, threonine 505, valine 683, and alanine 856 form a hydrogen bond with the 681‐arginine residue. The variant disrupts these bonds (Figure 1d) [24].

Analysis using mCSM, DynaMut2, and DDMut indicated a trend towards protein destabilization due to p.(Arg681Cys), with *ΔΔ*G values of − 1.459, − 1.01, and − 0.23 kcal/mol, respectively. The variant, located in the ATP binding domain of CH1, was shown by mCSM‐PP and mmCSM‐NA to destabilize protein–protein and protein–mRNA binding stability with *ΔΔ*G values of − 0.148 and − 3.38 kcal/mol, respectively (Figures 1c and 5 and Supporting Tables S4 and S5). Taken together, the analyses point towards loss‐of‐function of SNRNP200 due to the p.Arg681Cys variant.

Figure 5In silico evaluation of stability (ΔΔG) of protein structure and protein–protein and protein–RNA interaction affinities. (a) Results of mCSM‐Stability, DynaMut2, and DDMut for assessment stability (ΔΔG) and dynamic changes in proteins due to variants. (b) mCSM‐PPI and mmCSM‐NA analysis for the effects on protein–protein and protein–RNA interaction affinities upon variants. ΔΔG: a change in Gibbs free energy and a thermodynamic measure indicating the change in free energy stability of a protein upon variants with zero as cutoff (*Δ* *Δ*G < 0: destabilizing; *Δ* *Δ*G > 0 stabilizing).

**Figure:**
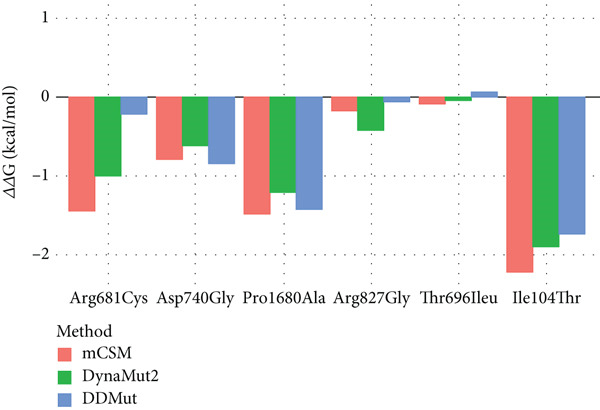
(a)

**Figure:**
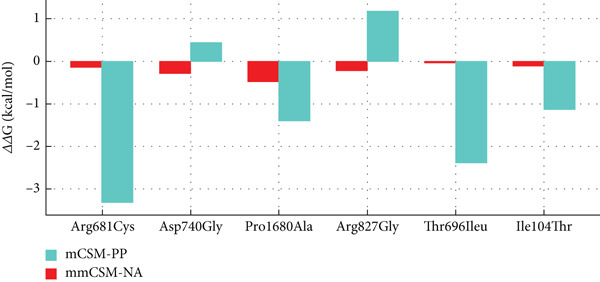
(b)

According to the ACMG guidelines, the variant was classified as pathogenic based on PM1 (moderate), as it is located in a critical, conserved functional domain; PM2 (moderate), due to its absence in population databases; PP3 (strong), supported by consistent predictions from computational tools indicating pathogenicity and structural destabilization; PP5 (strong), with reputable databases categorizing this variant as pathogenic; PM5 (strong), as a different missense change at the same residue is known to be pathogenic; PS4 (moderate), supported by statistical enrichment in affected individuals; and PP2 (moderate), given the high constraint against benign missense variation in the gene.

#### 3.1.2. Family 2

The proband (CLP‐1047‐3) had a left complete CLP. She had been adopted, and there was no information on her family history. Clinical examination at 4 years and 10 months of age noted normal growth and development. There was no sign of RP at 16 years and 5 months of age.

A heterozygous nucleotide substitution (NM_014014.5: *c.2219A>G*) on exon 17 of *SNRNP200* was identified. DNA from her biological parents was not available (Figure 1a). This change leads to a p.(Asp740Gly) substitution with a very high consensus score (19 of 20) for pathogenicity. It is not present in the gnomAD, deCAF, and RGC population databases, nor in the local Highlander database (Table 1). It is not reported in ClinVar, LOVD, or Geno2MP.

The variant is also located in the CH1 domain of *SNRNP200*, conserved through different species. It involves replacing the negatively charged aspartic acid with a neutral, smaller, more hydrophobic glycine (Figure 1b,c). Arpeggio analysis showed that Gly740 has fewer VdW clashes and more polar contacts than the wild type (Supporting Table S6).

Arg736 and Arg739 form salt bridges with the wild‐type Asp740 residue (Figure 1d). Therefore, the p.(Asp740Gly) substitution likely disturbs ionic interactions and impacts the interactions of the CH1 domain with pre‐mRNA processing factor 8 (PRP8), U5 small nuclear ribonucleoproteins (U5 snRNP), and U4/U6 small nuclear ribonucleoproteins (U4/U6 snRNP), which are crucial for spliceosome assembly and pre‐mRNA splicing [32]. Glycine is very flexible and can disturb the required rigidity of the protein at this position [24]. This was underscored by mCSM, DynaMut2, and DDMut analyses, showing variant trending towards protein destabilization with *ΔΔ*G values of − 0.802, − 0.62, and − 0.85 kcal/mol, respectively. Taken together, the SNRNP200 function and stability appear diminished. The mCSM‐PP predicted a slight destabilizing effect on protein‐protein affinity (*Δ* *Δ*G = −0.269 kcal/mol) while mmCSM‐NA demonstrated an increase in protein‐mRNA binding stability upon variant (*Δ* *Δ*G = 0.46 kcal/mol) (Figure 5, Supporting Tables S4 and S5).

According to ACMG guidelines, the variant was classified as likely pathogenic based on PM1 and PM2 (moderate), PP2 and PP3 (supporting).

#### 3.1.3. Family 3

The proband (CLP‐1250‐3) had a right complete CLP with an unremarkable family history. At follow‐up of 5 years and 5 months of age, she showed no sign of RP.

A heterozygous nucleotide substitution (NM_014014.5: *c.5038C>G*) on exon 36 of *SNRNP200* was identified in the proband and unaffected father, causing a p.(Pro1680Ala) substitution (consensus prediction score of 17 out of 20) (Figure 1a). Five alleles of this variant were reported in gnomAD (*N* = 1,611,356, AAF < 0.0001) and 10 alleles in RGC (out of 1,683,658 alleles, AAF < 0.0001). It was absent in deCAF and local Highlander database (Table 1). It was not reported in ClinVar, LOVD, or Geno2MP.

The variant at a conserved position in different species replaces proline with smaller alanine, disrupting necessary backbone conformation (Figure 1b). MCSM, DynaMut2, and DDMut showed that the variant causes protein destabilization (*ΔΔ*G values of − 1.49, − 1.22, and − 1.44 kcal/mol), affecting functionality and stability. Moreover, in silico modeling predicted an abnormal bond between mutant Ala1680 and Val1684, not present in the wild‐type, and MCSM‐PP and mmCSM‐NA suggested destabilization of protein–protein interactions (*ΔΔ*G − 0.492 kcal/mol) and protein–mRNA binding stability (*ΔΔ*G − 1.4 kcal/mol) (Figures 1 and 5, Supporting Tables S4 and S5).

Based on ACMG guidelines, the variant was categorized as likely pathogenic using PM1 (moderate), PM2 (moderate), PP2 (supporting), and PP3 (supporting).

### 3.2. SF3B1, SF3B2, and SF3B4

#### 3.2.1. Family 4

The proband (CLP‐1116‐3) had a posterior CP in the context of PRS. The proband’s parents showed no sign of PRS or CL/P. Mutations on *IRF6* and Collagen type II alpha 1 chain (*COL2A1*) were not found in the index person. At the age of 11 years and 9 months, no other syndrome, such as Stickler syndrome and myelodysplastic syndrome, was observed.

A heterozygous nucleotide change (NM_012433.4: *c.2479A>G*) on exon 17 of *SF3B1* was identified. Segregation analysis showed that the unaffected father was a carrier (Figure 2a). This variant causes a p.(Arg827Gly) substitution, with a consensus prediction of 18 out of 20. The variant is not reported in gnomAD, RGC, deCAF, or our Highlander local database (Table 1). It is not reported in ClinVar, LOVD, or Geno2MP.

The mutated residue is located in a conserved amino acid sequence (Figure 2b). The glycine is smaller, and more hydrophobic than the arginine. Arginine is positively charged, whereas glycine is neutral. Tyr830 and Arg865 form a salt bridge, which may be disturbed by the difference in charge at position 827. Glycine is also flexible and can disrupt the required rigidity of the protein (Figure 2d) [24]. Underscoring this, mCSM, DynaMut2, and DDMut demonstrated a trend towards protein destabilization, with *ΔΔ*G values of − 0.179, − 0.42, and − 0.06 kcal/mol, respectively. The substitution is located in an armadillo‐type fold or heat repeat 9 domain of *SF3B1* (Figure 2c), and Arg827 is normally in contact with residues of another heat repeat domain. The p.(Arg827Gly) variant could affect protein–protein contacts while enhancing protein–mRNA binding stability (mCSM‐PP: *Δ* *Δ*G = −0.216 kcal/mol, mmCSM‐NA: *Δ* *Δ*G = 1.19 kcal/mol) (Figure 5, Supporting Tables S4 and S5).

Based on ACMG guidelines, the variant was classified as likely pathogenic using PM1 (moderate), PM2 (moderate), PP2 (supporting), and PP3 (supporting).

#### 3.2.2. Family 5

The proband (CLP‐972‐3) has a complete cleft palate (CP). The parents did not have any sign of CL/P. At 14 years of age, the proband had no sign of craniofacial microsomia [33].

A heterozygous nucleotide change (NM_006842.3: *c.2087C>T*) was identified on exon 18 of *SF3B2* in the index individual and the unaffected mother, resulting in a p.(Thr696Ile) missense variant (Figure 3a). However, this alteration is located in the second nucleotide at the beginning of exon 18 (Figure 3b), and thus could cause an acceptor site loss (predicted by Mutation Taster, but not with Splice AI and CI‐SpliceAI) thereby disrupting splicing (Supporting Tables S2 and S3). However, functional RNA analysis could not be performed as patient RNA was not available. The majority of the algorithms used to predict pathogenicity cannot predict the impact on splicing. Five out of 20 algorithms predicted it as a damaging variant (Table S2). Only 7 such alleles were reported in gnomAD (*N* = 1,611,376), 13 alleles in RGC (out of 1,683,912 alleles, AAF < 0.0001), while it was absent in the deCAF database. The variant was seen once in the local database (Table 1). It was not reported in ClinVar, LOVD, or Geno2MP. The mutant residue (isoleucine) is bigger and more hydrophobic than the wild‐type residue (threonine). It may result in the decrease of hydrogen bonds, and VdW interactions and/or disrupt correct folding according to HOPE and Arpeggio [29]. MCSM and DynaMut2 suggested minor protein destabilization (*ΔΔ*G − 0.099 and − 0.04 kcal/mol, respectively), while DDMut indicated a slight increase in stability (*ΔΔ*G 0.06 kcal/mol). The variant may destabilize protein‐protein and protein–RNA interactions (*ΔΔ*G − 0.021 and − 2.375 kcal/mol) (Figure 5, Supporting Tables S4 and S5).

Based on the data presented here, including PM1 and PM2 (moderate), PP2 and PP3 (supporting), the variant is likely pathogenic under ACMG criteria.

#### 3.2.3. Family 6

The index (CLP‐1247‐3) individual presented with a posterior CP. The clinical examination of the parents was unremarkable. At the age of 5 years and 7 months, the proband showed no sign of Nager syndrome.

A trio analysis using ES identified a heterozygous nucleotide change (NM_005850.5: *c.311*T*>C*) on exon 3 of *SF3B4* in the proband and the unaffected father, causing a p.Ile104Thr with high consensus prediction (16 of 20) (Figure 4a). The variant is rare in gnomAD (14 alleles out of 1,461,888, AAF < 0.0001) and in RGC (17 alleles out of 1,683,912, AAF < 0.0001), and it is not reported in deCAF or local Highlander database (Table 1). It was reported once in ClinVar, and not in LOVD or Geno2MP.

The amino acid substitution, situated in a conserved position, involves a smaller, less hydrophobic threonine than the wild‐type isoleucine (Figure 4b). Arpeggio analysis revealed the reduced VdW clashes, and hydrophobic and carbonyl interactions with the mutant residue compared to wild type. This variant is located in the RNA recognition motif domain 2 (RRM 2) [24], which is important for binding RNA‐binding proteins and RNA molecules, such as U2 small nuclear RNA (snRNA) (Figure 4c). MCSM‐PP and mmCSM‐NA predicted destabilizing effects on protein–protein affinity and a significant decrease in protein–mRNA stability with a *ΔΔ*G of − 0.10 kcal/mol and *ΔΔ*G of − 1.13 kcal/mol, respectively. Further, mCSM, DynaMut2, and DDMut showed notable protein destabilization, with *ΔΔ*G values of − 2.22, − 1.91, and − 1.74 kcal/mol, respectively (Figures 4d and 5, Supporting Tables S4 and S5). With two moderate (PM1, PM2) and two supporting (PP2 and PP3) criteria, the variant is classified as likely pathogenic under ACMG guidelines.

## 4. Discussion

In the present study, six families were solved by identifying six rare variants in 26 spliceosomal candidate genes. These likely damaging variants include: three heterozygous missense variants in *SNRNP200* in three different NsOFCs index individuals, two missense variants in *SF3B1* and *SF3B4* in a posterior CP (in PRS), and a NsCP proband, respectively, and one missense/exonic splice site variant in *SF3B2* in one NsCP patient. These data suggest that variants in spliceosome genes may underlie NsOFC in up to 2.67% of patients in our cohort.

All the identified variants were either absent or rare in the large general population databases (PM2: moderate). Five of the six variants in *SNRNP200*, *SF3B1*, and *SF3B4* had high consensus prediction scores for pathogenicity, varying from 16 to 20 out of 20 (PP3: supporting). The variant in *SF3B2* was an exception due to the limited number of algorithms predicting splicing alterations. All variants had a high CADD score over 20 and one of the *SNRNP200* variants is already classified as pathogenic for RP [32]. Moreover, *SNRNP200*, *SF3B1*, *SF3B2*, and *SF3B4* have a high Z‐score in gnomAD for negative selection for missense variants (PP2: supporting). Finally, no other variant with such a high consensus score and rarity in population databases could be found for the six index individuals among the 544 known syndromic genes.

In the Franklin database, the variant in family 1 is predicted pathogenic, whereas the other 5 are categorized between VUS and likely pathogenic (LP), due to limited available data [34]. However, their other bioinformatic characteristics are in favor of LP, including high conservation of the positions in orthologous proteins through species, location in a functional domain (PM1: moderate), altered size and charge due to the substitution, and predicted altered 3D structure of the protein due to appearance of novel amino acid interactions. Moreover, stability and affinity changes suggest loss‐of‐function of the variant forms [26, 30]. In the context of CL/P, where incomplete penetrance is common, benign criteria such as BP2 (variant in an unaffected parent) were not applicable; thus, the variant meets the threshold for likely pathogenic.

Incomplete penetrance was observed, as four unaffected parents were carriers of the index‐variant. This is typical for genes causative of NsCL/P and NsCP. The pathophysiological basis is likely multifactorial, with a strong genetic predisposition. Importantly, non‐penetrance has also been previously documented for *SF3B4* (Nager syndrome) and *SNRPB* (cerebro–costo–mandibular syndrome), indicating that it is not a novel observation for spliceosome genes [8, 35, 36]. No families were identified that carried common variants in both the 550‐gene panel and the 26 spliceosome genes, precluding the assessment of digenic or oligogenic inheritance in this cohort. We do not currently have good tools to exploit the possibility that digenic (or even oligogenic) variants could play a role in the genetic predisposition. To this end, the DIDA database (http://dida.ibsquare.be) and ORVAL program may help in the future [37].

The spliceosome is a large RNA‐protein complex that recognizes introns in precursor messenger‐RNAs (pre‐mRNAs) and performs accurate splicing at the 5 ^′^ and 3 ^′^ splice‐sites. The major complex includes the U1, U2, U4, U5, and U6 snRNPs [11, 16]. *SNRNP200*, at the heart of U5 snRNP, interacts with the elongation factor Tu GTP binding domain containing 2 (*EFTUD2,* HGNC:30858) [38]. SF3b, an important part of U2 snRNP, consists of seven subunits, which are encoded by *SF3B1*, *SF3B2*, *SF3B3*, *SF3B4*, *SF3B5*, *SF3B6*, and *PHF5A* (PHD finger protein 5A). Interactions between SF3B4 and SF3B2, and SF3B4 and a region of the pre‐mRNA, occur upstream of the intronic branch site [36]. Thus, mutations in genes encoding spliceosome components can cause dysregulation of splicing, and subsequently cell‐ and/or tissue‐specific defects [33, 36, 39, 40].

The genetic disorders associated with spliceosome components and thereby sharing pathophysiological mechanisms affect especially the head and neck region including craniofacial defects, developmental delay, and retinitis pigmentosa [11, 13]. *SF3B2*, *SF3B4*, thioredoxin like 4A (*TXNL4A,* HGNC: 30551) small nuclear ribonucleoprotein polypeptides B And B1 (*SNRPB*, HGNC:11153) and *EFTUD2* mutations have been identified in craniofacial spliceosomopathies (SyOFC) and *SNRNP200* mutations have been recognized in RP [7–10, 38]. SF3B2 interacts with SF3B4, and mutations in either of them lead to diminished/altered/lack of cranial neural crest precursor formation and consequently to craniofacial cartilage defects [33, 39–42]. This supports an association between spliceosome mutations and impaired neural crest development in congenital craniofacial diseases [33, 36, 39, 40]. In zebrafish models, spliceosome gene deficiency impairs neural crest precursor formation [12, 33, 43], whereas recent mouse studies have shown that neural crest cells do form but subsequently undergo proliferation arrest and apoptosis, which also results in cleft palate and craniofacial malformations [44]. Together, these findings highlight that both impaired formation and later loss of viability of neural crest cells can converge on orofacial cleft phenotypes. In most craniofacial spliceosomopathies, the affected skeletal elements derive primarily from the neural crest, the embryonic cell population that makes an important contribution to the orofacial complex [11, 40].

We identified three heterozygous missense variants in SNRNP200. The Arg681Cys variant in the ATP binding domain of SNRNP200 is likely to cause a LoF by impairing ATP binding and hydrolysis, similar to other known mutations in this domain of RNA helicases. Arg681Cys has been reported in RP [32, 45, 46]. The CH1 domain primarily contributes to the shape and structural integrity of the helicase and its interactions with other core spliceosomal components. Variants in this domain, such as Asp740Gly, disrupt the structural conformation necessary for helicase activity. Both LoF variants, Arg681Cys and Asp740Gly, can lead to impaired RNA binding, disrupted protein interactions with other spliceosomal proteins, and disrupted RNA threading, such as the U4/U6 snRNA duplex, a series of ordered binding and rearrangement events during splicing that are critical for normal cellular function [45, 46]. In addition, the Pro1680Ala variant in the CH2 domain of SNRNP200, similar to the CH1 domain, can disrupt normal regulatory interactions, leading to a decrease in helicase activity or complete LoF. Based on our in‐silico analysis, the variant in CH2 also destabilizes the protein, decreasing its affinity for interactions with proteins and RNA, thus resulting in LoF [47].

Inhibition of *SNRNP200* in stem cells from the apical papilla decreased alkaline phosphatase (ALP) activity, mineralization, and expression of osteo‐/dentinogenic genes [48]. Moreover, knock‐down of *SNRNP200* repressed osteo‐/dentinogenic differentiation and cell proliferation by blocking cell cycle progression at the G2/M and S phase on dental tissue‐derived mesenchymal stem cells [48]. Therefore, the *SNRNP200* variants likely cause loss of functions such as osteo‐/dentinogenic differentiation and cell proliferation, as well as ordered binding and rearrangement events during splicing.

*SF3B1* is frequently mutated in myelodysplastic syndrome and various solid tumors [49]. SF3B1 promotes U2 snRNP stabilization at the branch site during spliceosome assembly. SF3B1, when mutated in conserved C‐terminal domain between the fourth and twelfth HEAT domain repeat, disrupts splice site recognition and binding affinity to spliceosome components, causing aberrant transcripts and degradation of mRNAs of target genes via nonsense‐mediated mRNA decay (NMD) [50, 51]. The p.Arg827Gly in the NSCL/P, located in heat repeat 9 domain, may cause similar disruption. In addition, *SF3B1* interaction with *ASXL1* (HGNC:18318), implicated in Bohring–Opitz syndrome (MIM:605039; another SyOFC), highlights the role of *SF3B1* in CL/P [11, 50]. The phenotypic variability suggests depending on the mutation type, location, and other genetic factors, the pathogenic variants in SF3B1 affect its function differently [51].

We identified a c.2087C>T variant in *SF3B2*. This alteration is within an exonic splice site, and likely causes an acceptor site loss and disrupts normal splicing, and/or NMD and haploinsufficiency [33]. De novo or transmitted haploinsufficiency‐causing *SF3B2* variants have been identified in 20 individuals from seven families with craniofacial microsomia (MIM:164210) with lateral oral cleft in some probands [33]. Thus, there seems to be a spectrum from NsCL/P to SyCLP and craniofacial macrosomia due to *SF3B2* pathogenic variants. Moreover, we found a heterozygous variant p.Ile104Thr on *SF3B4* in NsCL/P. The variant is located in the RRM 2 domain, important for pre‐mRNA binding. The variant likely disrupts this capacity. Moreover, the noncanonical SF3B4 function via binding to the bone morphogenetic protein (BMP) receptor BMPR1A could influence BMP‐mediated osteochondral cell differentiation [39].

This study has limitations. Functional validation was not conducted, so the impact of identified variants on gene function and cellular pathways remains elusive. In addition, blood sample of parents of family 1 and family 2 probands were not available, limiting insights into variant segregation and penetrance. RNA analysis of family 5 variant could not be performed, which limited our ability to confirm its splicing effects. Finally, the absence of long‐term follow‐up data limits insight into phenotypic variability, particularly in families 1, 2, and 3, as RP usually manifests only during childhood and adolescence. This also restricts our understanding of expressivity and health outcomes related to these variants. Future studies should address these gaps to provide a more comprehensive understanding.

## 5. Conclusion

In summary, mutations in genes encoding spliceosome components can cause dysregulated splicing, and subsequently cell‐ or tissue‐specific defects, resulting in a group of genetic disorders including retinitis pigmentosa, myelodysplastic syndromes, cancer, Nager syndrome, and craniofacial microsomia [11, 33, 50]. Our study reports rare, likely pathogenic variants in *SNRNP200*, *SF3B1*, *SF3B2*, and *SF3B4* as novel candidate genes for further exploration in NsCL/P and NsCP. It would be of interest to study the pathophysiological mechanisms of these spliceosome complex variants in vitro and/or in vivo, to identify the dysregulated target genes. Nonetheless, these spliceosomal genes, and eventually other spliceosome complex genes, should be given more focus in NsCL/P research. Studies will also be needed to determine the additional genetic and/or environmental factors that contribute to reduced penetrance and phenotypic heterogeneity [49]. Meanwhile, centers performing diagnostic testing for NsOFCs may consider including these spliceosomal genes within their diagnostic gene lists.

## Ethics Statement

The study procedure was endorsed by the ethical committee of the medical faculty at University of Louvain, Brussels, Belgium (2016/10 OCT/438 – BE403201629786).

## Consent

Participants signed an informed consent approved by institutional review boards.

## Disclosure

All authors have read and approved the final manuscript, and they agree to be accountable for all aspects of the work.

## Conflicts of Interest

The authors declare no conflicts of interest.

## Author Contributions

P.R., M.V.: conceptualization. P.R., M.V.: methodology. P.R., R.H., P.B.: software. P.R., E.P.: validation. P.R., E.P., N.R., A.G., B.B.: investigation. N.R., A.G., B.B., M.V.: resources. R.H., P.B.: data curation. P.R.: writing—original draft. P.R., E.P., R.H., N.R., A.G., B.B., M.V.: writing—review and editing. P.R., E.P., M.V.: visualization. M.V.: supervision. M.V.: project administration. M.V.: funding acquisition.

## Funding

This work was supported by the Fonds de la Recherche Scientifique – FNRS (T.0247.19 and CdR J.0228.20); the Walloon Region through the FRFS‐WELBIO strategic research programme (WELBIO‐CR‐2019C‐06); and a Pierre M. fellowship (all to M.V.). E.P. was supported by a FNRS Research Fellowship (ASP). P.B. is a Scientific Logistics Manager of the Genomics Platform of University of Louvain. We also acknowledge support from the National Lottery, Belgium; the Foundation against Cancer, Belgium (2010‐101); and the FNRS Equipment Grant (U.N035.17) for the Genetics Platform (to MV).

## Supporting Information

Additional supporting information can be found online in the Supporting Information section.

## Supporting information


**Supporting Information 1** Supporting Figure S1. Overview of the variant filtering process for sample analysis.

**Supporting Information 2** Supporting Table S1. List of 26 genes in the spliceosome complex chosen as candidate genes to find rare pathogenic variants in patients with NonSyndromic Orofacial Clefts.

**Supporting Information 3** Supporting Table S2. List of the twenty algorithms/tools used in Highlander to compute a consensus prediction for pathogenicity.

**Supporting Information 4** Supporting Table S3. List of the two algorithms/tools used for prediction of pathogenicity of splice site variant.

**Supporting Information 5** Supporting Table S4. In silico evaluation of stability (ΔΔG) and dynamic alterations in Three‐Dimensional protein structures due to variants.

**Supporting Information 6** Supporting Table S5. In silico analysis of variant‐induced changes in Protein‐Protein and Protein‐RNA interaction affinities.

**Supporting Information 7** Supporting Table S6. Analysis of local molecular interactions using Arpeggio: Comparative count of wild‐type and mutant residues.

**Supporting Information 8** Supporting Table S7. List of 544 candidate genes for oral clefts.

**Supporting Information 9** Supporting File S8. VariantValidator reports for all identified variants.

## Data Availability

All data are included in the manuscript and its supporting information files. In addition, the variants identified in this study have been submitted to the Leiden Open Variation Database (LOVD) at https://www.lovd.nl/, under the following submission numbers: links: Variant #0001048797: https://databases.lovd.nl/shared/variants/0001048797#00019945; Variant #0001048798: https://databases.lovd.nl/shared/variants/0001048798#00019945; Variant #0001048799: https://databases.lovd.nl/shared/variants/0001048799#00019945; Variant #0001048964: https://databases.lovd.nl/shared/variants/0001048964#00026017; Variant #0001048965: https://databases.lovd.nl/shared/variants/0001048965#00018767; and Variant #0001048966: https://databases.lovd.nl/shared/variants/0001048966#00018769.
