# Supplementary figures and images for: Likely Pathogenic/Pathogenic Variants in the Spliceosome Complex Genes SNRNP200, SF3B1, SF3B2, and SF3B4 Implicated in Nonsyndromic Orofacial Cleft
Source: Hum Mutat. 2025 Dec 14;2025:2991452. doi: 10.1155/humu/2991452 (PMC12714162; doi:10.1155/humu/2991452)

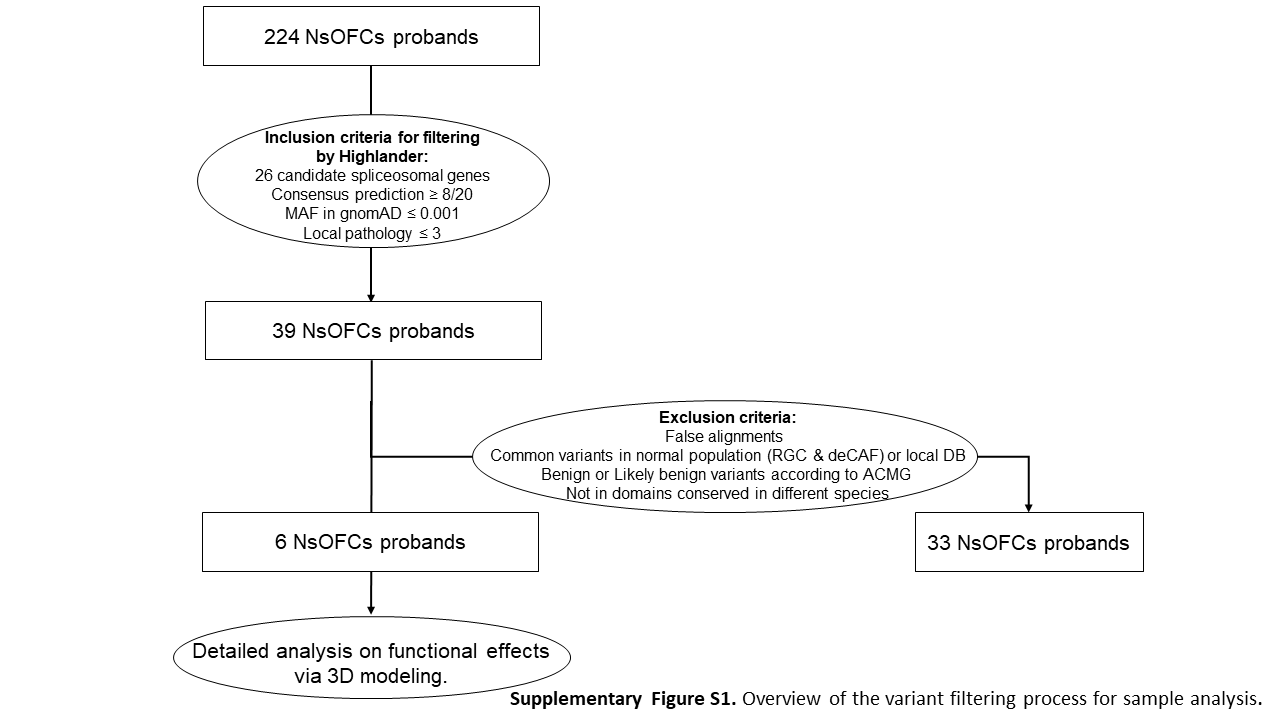

Supplement: Supplementary file 1 — Supporting Information 1 Supporting Figure S1. Overview of the variant filtering process for sample analysis. [file HUMU-2025-2991452-s005.tif]
