# Supplementary material for: Likely Pathogenic/Pathogenic Variants in the Spliceosome Complex Genes SNRNP200, SF3B1, SF3B2, and SF3B4 Implicated in Nonsyndromic Orofacial Cleft
Source: Hum Mutat. 2025 Dec 14;2025:2991452. doi: 10.1155/humu/2991452 (PMC12714162; doi:10.1155/humu/2991452)
Supplement: Supplementary file 2 — Supporting Information 2 Supporting Table S1. List of 26 genes in the spliceosome complex chosen as candidate genes to find rare pathogenic variants in patients with NonSyndromic Orofacial Clefts. [file HUMU-2025-2991452-s004.docx]

| *DDX23* | DEAD-Box helicase 23 |
| --- | --- |
| *EIF4A3* | Eukaryotic translation initiation factor 4A3 |
| *HNRNPR* | Heterogeneous nuclear ribonucleoprotein R |
| *PRPF3* | Pre-mRNA processing factor 3 |
| *PRPF4* | Pre-mRNA processing Factor 4 |
| *PRPF6* | pre-mRNA processing factor 6 |
| *PRPF8* | Pre-mRNA processing factor 8 |
| *PRPF31* | Pre-mRNA processing factor 31 |
| *SF1* | Splicing factor 1 |
| *SF3A1* | Splicing factor 3a subunit 1 |
| *SF3A3* | Splicing factor 3a subunit 3 |
| *SF3B1* | splicing factor 3b subunit 1 |
| *SF3B2* | splicing factor 3b subunit 2 |
| *SF3B3* | splicing factor 3b subunit 3 |
| *SF3B4* | splicing factor 3b subunit 4 |
| *SFMBT1* | Scm like with four Mbt domains 1 |
| *SFMBT2* | *Scm like with four Mbt domains 2* |
| *SNRNP200* | small nuclear ribonucleoprotein U5 200kDa subunit |
| *EFTUD2* | Elongation factor Tu GTP binding domain containing 2 |
| *TXNL4A* | Thioredoxin Like 4A |
| *SNRPE* | small nuclear ribonucleoprotein polypeptide E |
| *SNRPB* | small nuclear ribonucleoprotein polypeptide B |
| *PUF60* | Poly(U) Binding Splicing Factor 60 |
| *ZRSR2* | zinc finger CCCH-Type, RNA binding motif and serine/arginine rich 2 |
| *CWC27* | CWC27 Spliceosome Associated Cyclophilin |
| *RNU4ATAC* | RNA, U4atac Small Nuclear |

**Supplementary Table S1.** List of 26 genes in the spliceosome complex chosen as candidate genes to find rare pathogenic variants in patients with Non-Syndromic Orofacial Clefts [1-8].

References

1. Lehalle, D., et al., A review of craniofacial disorders caused by spliceosomal defects. Clinical genetics, 2015. 88(5): p. 405-415.
2. Griffin, C. and J.P. Saint‐Jeannet, Spliceosomopathies: diseases and mechanisms. Developmental Dynamics, 2020. 249(9): p. 1038-1046.
3. Stark, C., et al., BioGRID: a general repository for interaction datasets. Nucleic acids research, 2006. 34(suppl_1): p. D535-D539.
4. Will, C.L. and R. Lührmann, Spliceosome structure and function. Cold Spring Harbor perspectives in biology, 2011. 3(7): p. a003707.
5. Anna, A. and G. Monika, Splicing mutations in human genetic disorders: examples, detection, and confirmation. Journal of applied genetics, 2018. 59(3): p. 253-268.
6. Beauchamp MC, Alam SS, Kumar S, Jerome‐Majewska LA. Spliceosomopathies and neurocristopathies: Two sides of the same coin?. Developmental Dynamics. 2020 Aug;249(8):924-45.
7. Hannes, L., et al., Differential alternative splicing analysis links variation in ZRSR2 to a novel type of oral-facial-digital syndrome. Genet Med, 2024. 26(4): p. 101059.
8. Amudhavalli, S.M., et al., Novel blended SNRPE-related spliceosomopathy phenotype characterized by microcephaly and congenital atrichia. Am J Med Genet A, 2023. 191(5): p. 1425-1429.
