# Supplementary material for: Likely Pathogenic/Pathogenic Variants in the Spliceosome Complex Genes SNRNP200, SF3B1, SF3B2, and SF3B4 Implicated in Nonsyndromic Orofacial Cleft
Source: Hum Mutat. 2025 Dec 14;2025:2991452. doi: 10.1155/humu/2991452 (PMC12714162; doi:10.1155/humu/2991452)
Supplement: Supplementary file 3 — Supporting Information 3 Supporting Table S2. List of the twenty algorithms/tools used in Highlander to compute a consensus prediction for pathogenicity. [file HUMU-2025-2991452-s003.docx]

**Supplementary Table S2**: List of the twenty algorithms/tools used in Highlander to compute a consensus prediction for pathogenicity.

| **No** | **Algorithms/Tools** | **URL** | **References** |
| --- | --- | --- | --- |
| 1 | MutationTaster 2 | http://www.mutationtaster.org | doi.org/10.1038/nmeth.2890 |
| 2 | FATHMM v2.3 | http://fathmm.biocompute.org.uk | doi: 10.1093/bioinformatics/btt182 |
| 3 | Fathmm-XF | http://fathmm.biocompute.org.uk/fathmm-xf/ |  |
| 4 | Polyphen-2 v2.2.2, released Feb, 2012 | http://genetics.bwh.harvard.edu/pph2/ | doi: 10.1038/nmeth0410-248 |
| 5 | PROVEAN 1.1 ensembl 66, released Jan, 2015 | http://provean.jcvi.org/index.php | doi: 10.1093/bioinformatics/btv195 |
| 6 | SIFT4G 2.4, released Nov.1,2016 | http://sift.bii.a-star.edu.sg/sift4g/public/Homo_sapiens/ |  |
| 7 | MutationAssessor release 3 | http://mutationassessor.org/ | doi:10.1093/nar/gkr407 |
| 8 | M-CAP v1.3 | http://bejerano.stanford.edu/MCAP/ | doi.org/10.1038/ng.3703 |
| 9 | LRT released November, 2009 | http://www.genetics.wustl.edu/jflab/lrt_query.html | doi: 10.1101/gr.092619.109 |
| 10 | LIST-S2 release:2019_10 | https://precomputed.list-s2.msl.ubc.ca/ | doi:10.1093/nar/gkaa288 |
| 11 | Deogen2 | http://deogen2.mutaframe.com/ | doi: 10.1093/nar/gkx390  doi.org/10.1038/s41598-018-22531-2 |
| 12 | ClinPred | https://sites.google.com/site/clinpred/home | doi: 10.1016/j.ajhg.2018.08.005 |
| 13 | BayesDel v1 | http://bjfenglab.org/ | doi: 10.1002/humu.23158 |
| 14 | PrimateAI v0.2 | https://github.com/illumina/PrimateAI | doi.org/10.1038/s41588-018-0167-z |
| 15 | MetaSVM and MetaLR |  | doi.org/10.1093/hmg/ddu733 |
| 16 | CADD v1.6 | http://cadd.gs.washington.edu/ | doi: 10.1038/ng.2892 |
| 17 | VEST v4.0 | http://karchinlab.org/apps/appVest.html | doi:10.1186/1471-2164-14-S3-S3  doi:10.1002/humu.22911 |
| 18 | REVEL | http://sites.google.com/site/revelgenomics/ | dx.doi.org/10.1016/j.ajhg.2016.08.016 |
| 19 | MVP 1.0 | http://github.com/ShenLab/missense | doi.org/10.1038/s41467-020-20847-0 |
| 20 | Mutpred v1.2 | http://mutpred.mutdb.org | doi.org/10.1038/s41467-020-19669-x |
