# Supplementary material for: Likely Pathogenic/Pathogenic Variants in the Spliceosome Complex Genes SNRNP200, SF3B1, SF3B2, and SF3B4 Implicated in Nonsyndromic Orofacial Cleft
Source: Hum Mutat. 2025 Dec 14;2025:2991452. doi: 10.1155/humu/2991452 (PMC12714162; doi:10.1155/humu/2991452)
Supplement: Supplementary file 4 — Supporting Information 4 Supporting Table S3. List of the two algorithms/tools used for prediction of pathogenicity of splice site variant. [file HUMU-2025-2991452-s009.docx]

**Supplementary Table S3**: List of the two algorithms/tools used for prediction of pathogenicity of splice site variant.

| **No** | **Algorithms/Tools** | **URL** | **References** |
| --- | --- | --- | --- |
| 1 | SpliceAI | https://spliceailookup.broadinstitute.org/ | doi: 10.1016/j.cell.2018.12.015 |
| 2 | CI-SpliceAI | https://ci-spliceai.com/ | doi.org/10.1371/journal.pone.0269159 |
