# Supplementary material for: Likely Pathogenic/Pathogenic Variants in the Spliceosome Complex Genes SNRNP200, SF3B1, SF3B2, and SF3B4 Implicated in Nonsyndromic Orofacial Cleft
Source: Hum Mutat. 2025 Dec 14;2025:2991452. doi: 10.1155/humu/2991452 (PMC12714162; doi:10.1155/humu/2991452)
Supplement: Supplementary file 5 — Supporting Information 5 Supporting Table S4. In silico evaluation of stability (ΔΔG) and dynamic alterations in Three‐Dimensional protein structures due to variants. [file HUMU-2025-2991452-s002.docx]

**Supplementary Table S4.** In silico evaluation of stability (∆∆G) and dynamic alterations in Three-Dimensional protein structures due to variants

| **Gene Name** | **Variants** | **mCSM**  **ΔΔG^a^ kcol/mol)** | **DynaMut2**  **ΔΔG (kcol/mol)** | **DDMut ΔΔG(kcol/mol)** |
| --- | --- | --- | --- | --- |
| SNRNP200 | Arg681Cys | -1.459 | -1.01 | -0.23 |
| SNRNP200 | Asp740Gly | -0.802 | -0.62 | -0.85 |
| SNRNP200 | Pro1680Ala | -1.494 | -1.22 | -1.44 |
| SF3B1 | Arg827Gly | -0.179 | -0.42 | -0.06 |
| SF3B2 | Thr696Ileu | -0.099 | -0.04 | 0.06 |
| SF3B4 | Ile104Thr | -2.228 | -1.91 | -1.74 |

**^a^** Gibbs free energy (∆∆G)
