# Supplementary material for: Likely Pathogenic/Pathogenic Variants in the Spliceosome Complex Genes SNRNP200, SF3B1, SF3B2, and SF3B4 Implicated in Nonsyndromic Orofacial Cleft
Source: Hum Mutat. 2025 Dec 14;2025:2991452. doi: 10.1155/humu/2991452 (PMC12714162; doi:10.1155/humu/2991452)
Supplement: Supplementary file 6 — Supporting Information 6 Supporting Table S5. In silico analysis of variant‐induced changes in Protein‐Protein and Protein‐RNA interaction affinities. [file HUMU-2025-2991452-s007.docx]

**Supplementary Table S5.**  In silico analysis of variant-induced changes in Protein-Protein and Protein-RNA interaction affinities

| **Gene Name** | **variant** | **mCSM-PP ^a^ ΔΔG (kcol/mol)** | **mmCSM-NA ^b^ ΔΔG (kcol/mol)** |
| --- | --- | --- | --- |
| SNRNP200 | Arg681Cys | -0.148 | -3.38 |
| SNRNP200 | Asp740Gly | -0.269 | 0.46 |
| SNRNP200 | Pro1680Ala | -0.492 | -1.4 |
| SF3B1 | Arg827Gly | -0.216 | 1.19 |
| SF3B2 | Thr696Ileu | -0.021 | -2.375 |
| SF3B4 | Ile104Thr | -0.106 | -1.13 |

^a^ mCSM-PP: tools for prediction Protein-Protein interaction affinities upon variant

^b^ mmCSM-NA: tools for prediction Protein-RNA interaction affinities upon variant
