# Supplementary material for: Likely Pathogenic/Pathogenic Variants in the Spliceosome Complex Genes SNRNP200, SF3B1, SF3B2, and SF3B4 Implicated in Nonsyndromic Orofacial Cleft
Source: Hum Mutat. 2025 Dec 14;2025:2991452. doi: 10.1155/humu/2991452 (PMC12714162; doi:10.1155/humu/2991452)
Supplement: Supplementary file 7 — Supporting Information 7 Supporting Table S6. Analysis of local molecular interactions using Arpeggio: Comparative count of wild‐type and mutant residues. [file HUMU-2025-2991452-s006.docx]

**Supplementary Table S6.** Analysis of local molecular interactions using Arpeggio: Comparative count of wild-type and mutant residues.

| Subcategory | | Contact Type | *SNRNP200*  Arg681Cys Wt ^a^/ Mut ^b^ | *SNRNP200*  Asn740Gly Wt / Mut | *SNRNP200*  Pro1680Ala Wt / Mut | *SF3B1*  Arg827Gly Wt / Mut | *SF3B2*  Thr696Ile Wt / Mut | *SF3B4*  Ile104Thr Wt / Mut |
| --- | --- | --- | --- | --- | --- | --- | --- | --- |
| Mutually Exclusive Interactions | VdW^c^  interactions | | 2/2 | 3/1 | 1/1 | 1/1 | 1/0 | 4/0 |
|  | VdW clash interactions | | 5/2 | 3/2 | 2/2 | 0/0 | 0/0 | 4/3 |
|  | Proximal | | 121/53 | 77/54 | 59/48 | 35/33 | 34/37 | 90/83 |
| Feature Contacts | Polar contacts | | 7/3 | 4/6 | 3/3 | 2/2 | 3/2 | 2/2 |
|  | Weak polar contacts | | 3/1 | 2/2 | 1/1 | 1/0 | 1/1 | 3/3 |
|  | Hydrogen bonds | | 5/2 | 3/3 | 2/2 | 1/1 | 2/2 | 2/2 |
|  | Hydrophobic contacts | | 3/2 | 0/0 | 1/1 | 0/0 | 0/0 | 23/7 |
|  | Weak hydrogen bonds | | 1/0 | 2/1 | 2/2 | 1/1 | 2/1 | 1/1 |
|  | Carbonyl interactions | | 0/0 | 0/0 | 0/0 | 0/0 | 0/0 | 23/2 |

^a^ Wt: wild-type; ^b^ Mut: mutant residues; ^c^ VdW: van der Waals
