# Supplementary material for: Likely Pathogenic/Pathogenic Variants in the Spliceosome Complex Genes SNRNP200, SF3B1, SF3B2, and SF3B4 Implicated in Nonsyndromic Orofacial Cleft
Source: Hum Mutat. 2025 Dec 14;2025:2991452. doi: 10.1155/humu/2991452 (PMC12714162; doi:10.1155/humu/2991452)
Supplement: Supplementary file 8 — Supporting Information 8 Supporting Table S7. List of 544 candidate genes for oral clefts. [file HUMU-2025-2991452-s008.docx]

**Supplementary Table S7.** List of 544 candidate genes for oral clefts [1-4].

| **Acronym** | **Gene name** |
| --- | --- |
| *ABCA1* | ATP binding cassette subfamily A member 1 |
| *ABCB1* | ATP binding cassette subfamily B member 1 |
| *ACBD5* | acyl-CoA binding domain containing 5 |
| *ACTB* | actin beta |
| *ACTG1* | actin gamma 1 |
| *ACTN1* | actinin alpha 1 |
| *ADAM17* | ADAM metallopeptidase domain 17 |
| *ADH1A* | alcohol dehydrogenase 1A (class I), alpha polypeptide |
| *ADH1B* | alcohol dehydrogenase 1B (class I), beta polypeptide |
| *ADH1C* | alcohol dehydrogenase 1C (class I), gamma polypeptide |
| *ADH4* | alcohol dehydrogenase 4 (class II), pi polypeptide |
| *ADH5* | alcohol dehydrogenase 5 (class III), chi polypeptide |
| *AHCY* | adenosylhomocysteinase |
| *AHR* | aryl hydrocarbon receptor |
| *AIP* | aryl hydrocarbon receptor interacting protein |
| *ALDH1A1* | aldehyde dehydrogenase 1 family member A1 |
| *ALDH1L1* | aldehyde dehydrogenase 1 family member L1 |
| *ALG9* | ALG9 alpha-1,2-mannosyltransferase |
| *ALX1* | ALX homeobox 1 |
| *ALX3* | ALX homeobox 3 |
| *ALX4* | ALX homeobox 4 |
| *AMER1* | APC membrane recruitment protein 1 |
| *AMT* | aminomethyltransferase |
| *ANKRD11* | ankyrin repeat domain containing 11 |
| *APEX1* | apurinic/apyrimidinic endodeoxyribonuclease 1 |
| *APOA1* | apolipoprotein A1 |
| *APOA1BP* | Apolipoprotein A -1-Binding protein |
| *APOA5* | apolipoprotein A5 |
| *APOB* | apolipoprotein B |
| *APOC2* | apolipoprotein C2 |
| *APOE* | apolipoprotein E |
| *ARCN1* | archain 1 |
| *ARHGAP29* | Rho GTPase activating protein 29 |
| *ARHGAP31* | Rho GTPase activating protein 31 |
| *ARHGAP35* | Rho GTPase activating protein 35 |
| *ARNT* | aryl hydrocarbon receptor nuclear translocator |
| *ARNT2* | aryl hydrocarbon receptor nuclear translocator 2 |
| *ARVCF* | ARVCF delta catenin family member |
| *ARX* | aristaless related homeobox |
| *ASXL1* | ASXL transcriptional regulator 1 |
| *ATIC* | 5-aminoimidazole-4-carboxamide ribonucleotide formyltransferase/IMP cyclohydrolase |
| *ATR* | ATR serine/threonine kinase |
| *ATRX* | ATRX chromatin remodeler |
| *B3GALT6* | beta-1,3-galactosyltransferase 6 |
| *B3GALTL* | beta-3-Glucosyltransferase |
| *B3GAT3* | beta-1,3-glucuronyltransferase 3 |
| *B4GALT7* | beta-1,4-galactosyltransferase 7 |
| *BAMBI* | BMP and activin membrane bound inhibitor |
| *BARX1* | BARX homeobox 1 |
| *BARX2* | BARX homeobox 2 |
| *BCL3* | BCL3 transcription coactivator |
| *BCOR* | BCL6 corepressor |
| *BHMT* | betaine--homocysteine S-methyltransferase |
| *BHMT2* | betaine--homocysteine S-methyltransferase 2 |
| *BMP2* | bone morphogenetic protein 2 |
| *BMP4* | bone morphogenetic protein 4 |
| *BMP6* | bone morphogenetic protein 6 |
| *BMP10* | bone morphogenetic protein 10 |
| *BMPR1A* | bone morphogenetic protein receptor type 1A |
| *BMPR1B* | bone morphogenetic protein receptor type 1B |
| *BMPR2* | bone morphogenetic protein receptor type 2 |
| *BUB1B* | BUB1 mitotic checkpoint serine/threonine kinase B |
| *C1QTNF3* | C1q and TNF related 3 |
| *C2CD3* | C2 domain containing 3 centriole elongation regulator |
| *C5ORF42* | CHROMOSOME 5 OPEN READING FRAME 42 |
| *C21ORF2* | CHROMOSOME 21 OPEN READING FRAME 2 |
| *CANT1* | calcium activated nucleotidase 1 |
| *CASK* | calcium/calmodulin dependent serine protein kinase |
| *CASR* | calcium sensing receptor |
| *CBS* | cystathionine beta-synthase |
| *CC2D2A* | coiled-coil and C2 domain containing 2A |
| *CCDC6* | coiled-coil domain containing 6 |
| *CCR1* | C-C motif chemokine receptor 1 |
| *CCR6* | C-C motif chemokine receptor 6 |
| *CCT3* | chaperonin containing TCP1 subunit 3 |
| *CDC45* | cell division cycle 45 |
| *CDH1* | cadherin 1 |
| *CDH2* | cadherin 2 |
| *CDKN1C* | cyclin dependent kinase inhibitor 1C |
| *CDX4* | caudal type homeobox 4 |
| *CEACAM19* | CEA cell adhesion molecule 19 |
| *CEP120* | centrosomal protein 120 |
| *CETP* | cholesteryl ester transfer protein |
| *CFC1* | cripto, FRL-1, cryptic family 1 |
| *CHD1* | chromodomain helicase DNA binding protein 1 |
| *CHD7* | chromodomain helicase DNA binding protein 7 |
| *CHL1* | cell adhesion molecule L1 like |
| *CHRNA4* | cholinergic receptor nicotinic alpha 4 subunit |
| *CHRNG* | cholinergic receptor nicotinic gamma subunit |
| *CHST14* | carbohydrate sulfotransferase 14 |
| *CHSY1* | chondroitin sulfate synthase 1 |
| *CHUK* | component of inhibitor of nuclear factor kappa B kinase complex |
| *CKAP2L* | cytoskeleton associated protein 2 like |
| *CKM* | creatine kinase, M-type |
| *CLPTM1* | CLPTM1 regulator of GABA type A receptor forward trafficking |
| *COL2A1* | collagen type II alpha 1 chain |
| *COL9A1* | collagen type IX alpha 1 chain |
| *COL9A2* | collagen type IX alpha 2 chain |
| *COL9A3* | collagen type IX alpha 3 chain |
| *COL11A1* | collagen type XI alpha 1 chain |
| *COL11A2* | collagen type XI alpha 2 chain |
| *COLEC10* | collectin subfamily member 10 |
| *COLEC11* | collectin subfamily member 11 |
| *CRABP1* | cellular retinoic acid binding protein 1 |
| *CRELD1* | cysteine rich with EGF like domains 1 |
| *CSPP1* | centrosome and spindle pole associated protein 1 |
| *CTCF* | CCCTC-binding factor |
| *CTH* | cystathionine gamma-lyase |
| *CTNNB1* | catenin beta 1 |
| *CTNND1* | catenin delta 1 |
| *CUX2* | cut like homeobox 2 |
| *CYP1A1* | cytochrome P450 family 1 subfamily A member 1 |
| *CYP1A2* | cytochrome P450 family 1 subfamily A member 2 |
| *CYP1B1* | cytochrome P450 family 1 subfamily B member 1 |
| *CYP2D6* | cytochrome P450 family 2 subfamily D member 6 |
| *CYP2E1* | cytochrome P450 family 2 subfamily E member 1 |
| *CYP3A7* | cytochrome P450 family 3 subfamily A member 7 |
| *DDX3X* | DEAD-box helicase 3 X-linked |
| *DDX59* | DEAD-box helicase 59 |
| *DHCR7* | 7-dehydrocholesterol reductase |
| *DHCR24* | 24-dehydrocholesterol reductase |
| *DHFR* | dihydrofolate reductase |
| *DHODH* | dihydroorotate dehydrogenase (quinone) |
| *DIS3L2* | DIS3 like 3'-5' exoribonuclease 2 |
| *DKK1* | dickkopf WNT signaling pathway inhibitor 1 |
| *DLG1* | discs large MAGUK scaffold protein 1 |
| *DLL4* | delta like canonical Notch ligand 4 |
| *DLX1* | distal-less homeobox 1 |
| *DLX2* | distal-less homeobox 2 |
| *DLX3* | distal-less homeobox 3 |
| *DLX4* | distal-less homeobox 4 |
| *DLX5* | distal-less homeobox 5 |
| *DLX6* | distal-less homeobox 6 |
| *DMGDH* | dimethylglycine dehydrogenase |
| *DNMT3B* | DNA methyltransferase 3 beta |
| *DOCK6* | dedicator of cytokinesis 6 |
| *DSP* | desmoplakin |
| *DVL1* | dishevelled segment polarity protein 1 |
| *DVL3* | dishevelled segment polarity protein 3 |
| *DYNC2H1* | dynein cytoplasmic 2 heavy chain 1 |
| *DYNC2LI1* | dynein cytoplasmic 2 light intermediate chain 1 |
| *EBP* | EBP cholestenol delta-isomerase |
| *EDN1* | endothelin 1 |
| *EDNRA* | endothelin receptor type A |
| *EFNB1* | ephrin B1 |
| *EFTUD2* | elongation factor Tu GTP binding domain containing 2 |
| *EGF* | epidermal growth factor |
| *EGFR* | epidermal growth factor receptor |
| *EGR3* | early growth response 3 |
| *EIF2S3* | eukaryotic translation initiation factor 2 subunit gamma |
| *EIF4A3* | eukaryotic translation initiation factor 4A3 |
| *EMX2* | empty spiracles homeobox 2 |
| *EOGT* | EGF domain specific O-linked N-acetylglucosamine transferase |
| *EPG5* | ectopic P-granules 5 autophagy tethering factor |
| *EPHB2* | EPH receptor B2 |
| *EPHB3* | EPH receptor B3 |
| *EPHX1* | epoxide hydrolase 1 |
| *EPS15* | epidermal growth factor receptor pathway substrate 15 |
| *EPT1* | ETHANOLAMINEPHOSPHOTRANSFERASE |
| *ERCC2* | ERCC excision repair 2, TFIIH core complex helicase subunit |
| *ERCC4* | ERCC excision repair 4, endonuclease catalytic subunit |
| *ESCO2* | establishment of sister chromatid cohesion N-acetyltransferase 2 |
| *ESR1* | estrogen receptor 1 |
| *ESR2* | estrogen receptor 2 |
| *ESRP2* | epithelial splicing regulatory protein 2 |
| *ESRRB* | estrogen related receptor beta |
| *ETV5* | ETS variant transcription factor 5 |
| *EVC* | EvC ciliary complex subunit 1 |
| *EVC2* | EvC ciliary complex subunit 2 |
| *EYA1* | EYA transcriptional coactivator and phosphatase 1 |
| *F13A1* | coagulation factor XIII A chain |
| *FAM20C* | FAM20C golgi associated secretory pathway kinase |
| *FAM111A* | FAM111 trypsin like peptidase A |
| *FANCL* | FA complementation group L |
| *FBXO11* | F-box protein 11 |
| *FGD1* | FYVE, RhoGEF and PH domain containing 1 |
| *FGF1* | fibroblast growth factor 1 |
| *FGF2* | fibroblast growth factor 2 |
| *FGF4* | fibroblast growth factor 4 |
| *FGF5* | fibroblast growth factor 5 |
| *FGF7* | fibroblast growth factor 7 |
| *FGF8* | fibroblast growth factor 8 |
| *FGF9* | fibroblast growth factor 9 |
| *FGF10* | fibroblast growth factor 10 |
| *FGF12* | fibroblast growth factor 12 |
| *FGFBP1* | fibroblast growth factor binding protein 1 |
| *FGFR1* | fibroblast growth factor receptor 1 |
| *FGFR2* | fibroblast growth factor receptor 2 |
| *FGFR3* | fibroblast growth factor receptor 3 |
| *FGFR4* | fibroblast growth factor receptor 4 |
| *FLNA* | filamin A |
| *FLNB* | filamin B |
| *FOLH1* | folate hydrolase 1 |
| *FOLR1* | folate receptor alpha |
| *FOLR2* | folate receptor beta |
| *FOLR3* | folate receptor gamma |
| *FOXC2* | forkhead box C2 |
| *FOXE1* | forkhead box E1 |
| *FOXF2* | forkhead box F2 |
| *FOXH1* | forkhead box H1 |
| *FOXN1* | forkhead box N1 |
| *FOXN3* | forkhead box N3 |
| *FOXP2* | forkhead box P2 |
| *FRAS1* | Fraser extracellular matrix complex subunit 1 |
| *FREM2* | FRAS1 related extracellular matrix 2 |
| *FSCN1* | fascin actin-bundling protein 1 |
| *FST* | follistatin |
| *FTCD* | formimidoyltransferase cyclodeaminase |
| *FTO* | FTO alpha-ketoglutarate dependent dioxygenase |
| *FZD1* | frizzled class receptor 1 |
| *FZD2* | frizzled class receptor 2 |
| *FZD4* | frizzled class receptor 4 |
| *FZD7* | frizzled class receptor 7 |
| *FZD8* | frizzled class receptor 8 |
| *FZD10* | frizzled class receptor 10 |
| *GABBR2* | gamma-aminobutyric acid type B receptor subunit 2 |
| *GABRB3* | gamma-aminobutyric acid type A receptor subunit beta3 |
| *GAD1* | glutamate decarboxylase 1 |
| *GAD2* | glutamate decarboxylase 2 |
| *GART* | phosphoribosylglycinamide formyltransferase, phosphoribosylglycinamide synthetase, phosphoribosylaminoimidazole synthetase |
| *GATA3* | GATA binding protein 3 |
| *GATA6* | GATA binding protein 6 |
| *GDF1* | growth differentiation factor 1 |
| *GJA1* | gap junction protein alpha 1 |
| *GJB2* | gap junction protein beta 2 |
| *GLI2* | GLI family zinc finger 2 |
| *GLI3* | GLI family zinc finger 3 |
| *GMNN* | geminin DNA replication inhibitor |
| *GNAI3* | G protein subunit alpha i3 |
| *GNB1* | G protein subunit beta 1 |
| *GNMT* | glycine N-methyltransferase |
| *GPC3* | glypican 3 |
| *GRHL3* | grainyhead like transcription factor 3 |
| *GRIP1* | glutamate receptor interacting protein 1 |
| *GSTA4* | glutathione S-transferase alpha 4 |
| *GSTM1* | glutathione S-transferase mu 1 |
| *GSTM3* | glutathione S-transferase mu 3 |
| *GSTP1* | glutathione S-transferase pi 1 |
| *GSTT1* | glutathione S-transferase theta 1 |
| *GYPE* | glycophorin E (MNS blood group) |
| *HAND2* | heart and neural crest derivatives expressed 2 |
| *HDAC8* | histone deacetylase 8 |
| *HIC1* | HIC ZBTB transcriptional repressor 1 |
| *HIF1A* | hypoxia inducible factor 1 subunit alpha |
| *HOXA2* | homeobox A2 |
| *HOXA7* | homeobox A7 |
| *HOXB6* | homeobox B6 |
| *HSP90AA1* | heat shock protein 90 alpha family class A member 1 |
| *HYAL1* | hyaluronidase 1 |
| *HYLS1* | HYLS1 centriolar and ciliogenesis associated |
| *ICK* | INTESTINAL CELL KINASE |
| *IDH1* | isocitrate dehydrogenase (NADP(+)) 1 |
| *IFNK* | interferon kappa |
| *IFT52* | intraflagellar transport 52 |
| *IFT80* | intraflagellar transport 80 |
| *IFT81* | intraflagellar transport 81 |
| *IFT122* | intraflagellar transport 122 |
| *IFT140* | intraflagellar transport 140 |
| *IFT172* | intraflagellar transport 172 |
| *IKBKE* | inhibitor of nuclear factor kappa B kinase subunit epsilon |
| *IMPAD1* | INOSITOL MONOPHOSPHATASE DOMAIN-CONTAINING PROTEIN 1 |
| *INHBA* | inhibin subunit beta A |
| *INHBB* | inhibin subunit beta B |
| *INTS1* | integrator complex subunit 1 |
| *INTU* | inturned planar cell polarity protein |
| *IRF6* | interferon regulatory factor 6 |
| *IRF9* | interferon regulatory factor 9 |
| *ITGB3* | integrin subunit beta 3 |
| *JAG1* | JAGGED 1 |
| *JAG2* | jagged canonical Notch ligand 2 |
| *KANSL1* | KAT8 regulatory NSL complex subunit 1 |
| *KAT6A* | lysine acetyltransferase 6A |
| *KAT6B* | lysine acetyltransferase 6B |
| *KCNJ2* | potassium inwardly rectifying channel subfamily J member 2 |
| *KDM1A* | lysine demethylase 1A |
| *KDM6A* | lysine demethylase 6A |
| *KIAA0196* | STRUMPELLIN |
| *KIAA0586* | KIAA0586 |
| *KIAA1279* | KINESIN-BINDING PROTEIN |
| *KIF7* | kinesin family member 7 |
| *KIF22* | kinesin family member 22 |
| *KLHL4* | kelch like family member 4 |
| *KMT2D* | lysine methyltransferase 2D |
| *KREMEN1* | kringle containing transmembrane protein 1 |
| *KRT14* | keratin 14 |
| *KRT18* | keratin 18 |
| *L1CAM* | L1 cell adhesion molecule |
| *LCAT* | lecithin-cholesterol acyltransferase |
| *LDLR* | low density lipoprotein receptor |
| *LEF1* | lymphoid enhancer binding factor 1 |
| *LEFTY2* | left-right determination factor 2 |
| *LHX8* | LIM homeobox 8 |
| *LIMK1* | LIM domain kinase 1 |
| *LIPC* | lipase C, hepatic type |
| *LMNA* | lamin A/C |
| *LMX1B* | LIM homeobox transcription factor 1 beta |
| *LOR* | LORICRIN |
| *LPL* | lipoprotein lipase |
| *MAP3K7* | mitogen-activated protein kinase kinase kinase 7 |
| *MAPRE2* | microtubule associated protein RP/EB family member 2 |
| *MARK4* | microtubule affinity regulating kinase 4 |
| *MASP1* | MBL associated serine protease 1 |
| *MAT1A* | methionine adenosyltransferase 1A |
| *MAT2A* | methionine adenosyltransferase 2A |
| *MAT2B* | methionine adenosyltransferase 2 non-catalytic beta subunit |
| *MBTPS2* | membrane bound transcription factor peptidase, site 2 |
| *MECOM* | MDS1 and EVI1 complex locus |
| *MED12* | mediator complex subunit 12 |
| *MED13L* | mediator complex subunit 13L |
| *MED25* | mediator complex subunit 25 |
| *MEIS2* | Meis homeobox 2 |
| *MEOX1* | mesenchyme homeobox 1 |
| *METTL23* | methyltransferase 23, arginine |
| *MID1* | midline 1 |
| *MKS1* | MKS transition zone complex subunit 1 |
| *MKX* | mohawk homeobox |
| *MMEL1* | membrane metalloendopeptidase like 1 |
| *MMP2* | matrix metallopeptidase 2 |
| *MMP13* | matrix metallopeptidase 13 |
| *MMP14* | matrix metallopeptidase 14 |
| *MSC* | musculin |
| *MSX1* | msh homeobox 1 |
| *MSX2* | msh homeobox 2 |
| *MT1A* | metallothionein 1A |
| *MT4* | metallothionein 4 |
| *MTHFD1* | methylenetetrahydrofolate dehydrogenase, cyclohydrolase and formyltetrahydrofolate synthetase 1 |
| *MTHFD2* | methylenetetrahydrofolate dehydrogenase (NADP+ dependent) 2, methenyltetrahydrofolate cyclohydrolase |
| *MTHFR* | methylenetetrahydrofolate reductase |
| *MTHFS* | methenyltetrahydrofolate synthetase |
| *MTR* | 5-methyltetrahydrofolate-homocysteine methyltransferase |
| *MTRR* | 5-methyltetrahydrofolate-homocysteine methyltransferase reductase |
| *MYL2* | myosin light chain 2 |
| *NAT1* | N-ACETYLTRANSFERASE 1 |
| *NAT2* | N-ACETYLTRANSFERASE 2 |
| *NBN* | nibrin |
| *NEDD4L* | NEDD4 like E3 ubiquitin protein ligase |
| *NEK1* | NIMA related kinase 1 |
| *NIPBL* | NIPBL cohesin loading factor |
| *NKX2-5* | NK2 homeobox 5 |
| *NKX2-6* | NK2 homeobox 6 |
| *NNMT* | nicotinamide N-methyltransferase |
| *NOTCH1* | notch receptor 1 |
| *NOTCH3* | notch receptor 3 |
| *NQO1* | NAD(P)H quinone dehydrogenase 1 |
| *NR3C1* | nuclear receptor subfamily 3 group C member 1 |
| *NRXN2* | neurexin 2 |
| *NSDHL* | NAD(P) dependent steroid dehydrogenase-like |
| *OFD1* | OFD1 centriole and centriolar satellite protein |
| *OGG1* | 8-oxoguanine DNA glycosylase |
| *OSR2* | odd-skipped related transciption factor 2 |
| *PAFAH1B1* | platelet activating factor acetylhydrolase 1b regulatory subunit 1 |
| *PAX3* | paired box 3 |
| *PAX8* | paired box 8 |
| *PAX9* | paired box 9 |
| *PCBD2* | pterin-4 alpha-carbinolamine dehydratase 2 |
| *PDGFC* | platelet derived growth factor C |
| *PDGFRA* | platelet derived growth factor receptor alpha |
| *PEX7* | peroxisomal biogenesis factor 7 |
| *PGAP2* | post-GPI attachment to proteins 2 |
| *PGM1* | phosphoglucomutase 1 |
| *PHF8* | PHD finger protein 8 |
| *PHGDH* | phosphoglycerate dehydrogenase |
| *PIEZO2* | piezo type mechanosensitive ion channel component 2 |
| *PIGA* | phosphatidylinositol glycan anchor biosynthesis class A |
| *PIGL* | phosphatidylinositol glycan anchor biosynthesis class L |
| *PIGN* | phosphatidylinositol glycan anchor biosynthesis class N |
| *PIGV* | phosphatidylinositol glycan anchor biosynthesis class V |
| *PIK3R2* | phosphoinositide-3-kinase regulatory subunit 2 |
| *PIPOX* | pipecolic acid and sarcosine oxidase |
| *PITX1* | paired like homeodomain 1 |
| *PITX2* | paired like homeodomain 2 |
| *PKP1* | plakophilin 1 |
| *PLCB4* | phospholipase C beta 4 |
| *PLEKHA5* | pleckstrin homology domain containing A5 |
| *PLEKHA7* | pleckstrin homology domain containing A7 |
| *POLR1A* | RNA polymerase I subunit A |
| *POLR1C* | RNA polymerase I and III subunit C |
| *POLR1D* | RNA polymerase I and III subunit D |
| *POMT1* | protein O-mannosyltransferase 1 |
| *POMT2* | protein O-mannosyltransferase 2 |
| *PON1* | paraoxonase 1 |
| *PORCN* | porcupine O-acyltransferase |
| *PQBP1* | polyglutamine binding protein 1 |
| *PRDM16* | PR/SET domain 16 |
| *PRRX1* | paired related homeobox 1 |
| *PRRX2* | paired related homeobox 2 |
| *PSAT1* | phosphoserine aminotransferase 1 |
| *PTCH1* | patched 1 |
| *PTCH2* | patched 2 |
| *PTDSS1* | phosphatidylserine synthase 1 |
| *PTEN* | phosphatase and tensin homolog |
| *PTPN11* | protein tyrosine phosphatase non-receptor type 11 |
| *PVR* | PVR cell adhesion molecule |
| *PVRL1* | POLIOVIRUS RECEPTOR-LIKE 1 |
| *PVRL2* | POLIOVIRUS RECEPTOR-LIKE 2 |
| *PVRL3* | POLIOVIRUS RECEPTOR-LIKE 3 |
| *RAI1* | retinoic acid induced 1 |
| *RARA* | retinoic acid receptor alpha |
| *RARB* | retinoic acid receptor beta |
| *RARG* | retinoic acid receptor gamma |
| *RBM8A* | RNA binding motif protein 8A |
| *RBM10* | RNA binding motif protein 10 |
| *RBPJ* | recombination signal binding protein for immunoglobulin kappa J region |
| *RECQL4* | RecQ like helicase 4 |
| *RET* | ret proto-oncogene |
| *RFC1* | replication factor C subunit 1 |
| *RIPK3* | receptor interacting serine/threonine kinase 3 |
| *ROR1* | receptor tyrosine kinase like orphan receptor 1 |
| *ROR2* | receptor tyrosine kinase like orphan receptor 2 |
| *RPL5* | ribosomal protein L5 |
| *RPL11* | ribosomal protein L11 |
| *RPS17* | ribosomal protein S17 |
| *RPS19* | ribosomal protein S19 |
| *RPS26* | ribosomal protein S26 |
| *RPS28* | ribosomal protein S28 |
| *RSPO2* | R-spondin 2 |
| *RUNX2* | RUNX family transcription factor 2 |
| *RXRG* | retinoid X receptor gamma |
| *RYK* | receptor like tyrosine kinase |
| *RYR1* | ryanodine receptor 1 |
| *SALL1* | spalt like transcription factor 1 |
| *SALL2* | spalt like transcription factor 2 |
| *SALL3* | spalt like transcription factor 3 |
| *SALL4* | spalt like transcription factor 4 |
| *SAR1A* | secretion associated Ras related GTPase 1A |
| *SATB2* | SATB homeobox 2 |
| *SC5D* | sterol-C5-desaturase |
| *SCARF2* | scavenger receptor class F member 2 |
| *SCD5* | stearoyl-CoA desaturase 5 |
| *SEC23A* | SEC23 homolog A, COPII coat complex component |
| *SET* | SET nuclear proto-oncogene |
| *SF3B4* | splicing factor 3b subunit 4 |
| *SHFM3* | SPLIT-HAND/FOOT MALFORMATION 3 |
| *SHH* | sonic hedgehog signaling molecule |
| *SHMT1* | serine hydroxymethyltransferase 1 |
| *SHMT2* | serine hydroxymethyltransferase 2 |
| *SIX1* | SIX homeobox 1 |
| *SIX3* | SIX homeobox 3 |
| *SIX5* | SIX homeobox 5 |
| *SKI* | SKI proto-oncogene |
| *SLC7A11* | solute carrier family 7 member 11 |
| *SLC26A2* | solute carrier family 26 member 2 |
| *SMAD1* | SMAD family member 1 |
| *SMAD2* | SMAD family member 2 |
| *SMAD3* | SMAD family member 3 |
| *SMAD4* | SMAD family member 4 |
| *SMC1A* | structural maintenance of chromosomes 1A |
| *SMC3* | structural maintenance of chromosomes 3 |
| *SMG9* | SMG9 nonsense mediated mRNA decay factor |
| *SMOC1* | SPARC related modular calcium binding 1 |
| *SMS* | spermine synthase |
| *SNAI1* | snail family transcriptional repressor 1 |
| *SNAI2* | snail family transcriptional repressor 2 |
| *SNRPB* | small nuclear ribonucleoprotein polypeptides B and B1 |
| *SNX3* | sorting nexin 3 |
| *SON* | SON DNA and RNA binding protein |
| *SOX1* | SRY-box transcription factor 1 |
| *SOX2* | SRY-box transcription factor 2 |
| *SOX5* | SRY-box transcription factor 5 |
| *SOX9* | SRY-box transcription factor 9 |
| *SP8* | Sp8 transcription factor |
| *SPAM1* | sperm adhesion molecule 1 |
| *SPECC1L* | sperm antigen with calponin homology and coiled-coil domains 1 like |
| *SPP1* | secreted phosphoprotein 1 |
| *SPPL3* | signal peptide peptidase like 3 |
| *SPRY2* | sprouty RTK signaling antagonist 2 |
| *SPTLC1* | serine palmitoyltransferase long chain base subunit 1 |
| *STAMBP* | STAM binding protein |
| *STAT3* | signal transducer and activator of transcription 3 |
| *STIL* | STIL centriolar assembly protein |
| *STRA6* | signaling receptor and transporter of retinol STRA6 |
| *STX18* | syntaxin 18 |
| *STXBP1* | syntaxin binding protein 1 |
| *SULT1A1* | sulfotransferase family 1A member 1 |
| *SUMO1* | small ubiquitin like modifier 1 |
| *TBX1* | T-box transcription factor 1 |
| *TBX4* | T-box transcription factor 4 |
| *TBX10* | T-box transcription factor 10 |
| *TBX15* | T-box transcription factor 15 |
| *TBX21* | T-box transcription factor 21 |
| *TBX22* | T-box transcription factor 22 |
| *TCF21* | transcription factor 21 |
| *TCOF1* | treacle ribosome biogenesis factor 1 |
| *TCTEX1D2* | TCTEX1 DOMAIN-CONTAINING PROTEIN 2 |
| *TCTN3* | tectonic family member 3 |
| *TELO2* | telomere maintenance 2 |
| *TFAP2A* | transcription factor AP-2 alpha |
| *TFAP2B* | transcription factor AP-2 beta |
| *TGDS* | TDP-glucose 4,6-dehydratase |
| *TGFB1* | transforming growth factor beta 1 |
| *TGFB2* | transforming growth factor beta 2 |
| *TGFB3* | transforming growth factor beta 3 |
| *TGFBR1* | transforming growth factor beta receptor 1 |
| *TGFBR2* | transforming growth factor beta receptor 2 |
| *TGFBR3* | transforming growth factor beta receptor 3 |
| *TGIF1* | TGFB induced factor homeobox 1 |
| *THRB* | thyroid hormone receptor beta |
| *TIMP2* | TIMP metallopeptidase inhibitor 2 |
| *TMCO1* | transmembrane and coiled-coil domains 1 |
| *TMEM8C* | TRANSMEMBRANE PROTEIN 8C |
| *TNFRSF10B* | TNF receptor superfamily member 10b |
| *TNNT3* | troponin T3, fast skeletal type |
| *TP63* | tumor protein p63 |
| *TRAPPC9* | trafficking protein particle complex subunit 9 |
| *TRIM37* | tripartite motif containing 37 |
| *TRPS1* | transcriptional repressor GATA binding 1 |
| *TSR2* | TSR2 ribosome maturation factor |
| *TTC21B* | tetratricopeptide repeat domain 21B |
| *TUBB* | tubulin beta class I |
| *TULP3* | TUB like protein 3 |
| *TWIST1* | twist family bHLH transcription factor 1 |
| *TWIST2* | twist family bHLH transcription factor 2 |
| *TXNL4A* | thioredoxin like 4A |
| *TYMS* | thymidylate synthetase |
| *UBB* | ubiquitin B |
| *UFD1L* | UBIQUITIN FUSION DEGRADATION 1-LIKE |
| *UGT1A7* | UDP glucuronosyltransferase family 1 member A7 |
| *UQCC2* | ubiquinol-cytochrome c reductase complex assembly factor 2 |
| *USP9X* | ubiquitin specific peptidase 9 X-linked |
| *VAX1* | ventral anterior homeobox 1 |
| *VCL* | vinculin |
| *WDR19* | WD repeat domain 19 |
| *WDR34* | WD REPEAT-CONTAINING PROTEIN 34 |
| *WDR35* | WD repeat domain 35 |
| *WDR60* | WD REPEAT-CONTAINING PROTEIN 60 |
| *WHSC1* | WOLF-HIRSCHHORN SYNDROME CANDIDATE 1 |
| *WNT3* | Wnt family member 3 |
| *WNT3A* | Wnt family member 3A |
| *WNT4* | Wnt family member 4 |
| *WNT5A* | Wnt family member 5A |
| *WNT6* | Wnt family member 6 |
| *WNT7B* | Wnt family member 7B |
| *WNT9B* | Wnt family member 9B |
| *XRCC1* | X-ray repair cross complementing 1 |
| *XRCC3* | X-ray repair cross complementing 3 |
| *XYLT1* | xylosyltransferase 1 |
| *YAP1* | Yes1 associated transcriptional regulator |
| *ZBTB24* | zinc finger and BTB domain containing 24 |
| *ZEB2* | zinc finger E-box binding homeobox 2 |
| *ZIC2* | Zic family member 2 |
| *ZIC3* | Zic family member 3 |
| *ZMPSTE24* | zinc metallopeptidase STE24 |
| *ZNF189* | zinc finger protein 189 |
| *ZSWIM6* | zinc finger SWIM-type containing 6 |

**References**

1. Jugessur, A., et al., *Genetic determinants of facial clefting: analysis of 357 candidate genes using two national cleft studies from Scandinavia.* PLoS One, 2009. **4**(4): p. e5385.

2. Piñero, J., et al., *The DisGeNET knowledge platform for disease genomics: 2019 update.* Nucleic Acids Research, 2019. **48**(D1): p. D845-D855.

3. Reynolds, K., et al., *Wnt signaling in orofacial clefts: crosstalk, pathogenesis and models.* Dis Model Mech, 2019. **12**(2).

4. Nasreddine, G., J. El Hajj, and M. Ghassibe-Sabbagh, *Orofacial clefts embryology, classification, epidemiology, and genetics.* Mutation Research/Reviews in Mutation Research, 2021. **787**: p. 108373.
