# Supplementary material for: Likely Pathogenic/Pathogenic Variants in the Spliceosome Complex Genes SNRNP200, SF3B1, SF3B2, and SF3B4 Implicated in Nonsyndromic Orofacial Cleft
Source: Hum Mutat. 2025 Dec 14;2025:2991452. doi: 10.1155/humu/2991452 (PMC12714162; doi:10.1155/humu/2991452)
Supplement: Supplementary file 9 — Supporting Information 9 Supporting File S8. VariantValidator reports for all identified variants. [file HUMU-2025-2991452-s001.pdf]

Supplementary file S8. VariantValidator reports for all identified variants.

A. Variant description of family 1 by VariantValidator

VariantValidator

Submitted Variant

NM_014014.5:c.2041C>T

- Selected genome build: GRCh38
- Map location: 2q11.2
- Transcript Flag: MANE Select
- CCDS ID: CCDS2020.1

Versions

- VariantValidator 2.2.1.dev341+g0388933
- vv_hgvs 2.2.0
- VVDb vvdb_2023_8
- VVTA vvta_2023_05
- VVSeqRepo VV_SR_2023_05/master

Recommended Variant Descriptions

1. HGVS guidelines recommend using genomic and transcript descriptions in all publications
2. Use of the three- or one-letter amino acid alphabet is optional, but three-letter is recommended

Genomic descriptions

**Reference Sequence Type**	**Variant Description**
Chromosomal GRCh37	NC_000002.11:g.96958829G>A
Chromosomal GRCh38	NC_000002.12:g.96293091G>A

Transcript and protein descriptions

**Reference Sequence Type**	**Variant Description**
Transcript	NM_014014.5:c.2041C>T
Protein three letter code	NP_054733.2:p.(Arg681Cys)
Protein single letter code	NP_054733.2:p.(R681C)

Gene Information

**Attribute**	**Identifier**	**Source**
Symbol	SNRNP200	HGNC
Name	small nuclear ribonucleoprotein U5 subunit 200	HGNC
HGNC ID	HGNC:30859	HGNC

MANCHESTER 1824
The University of Manchester

Copyright © 2016-2023 VariantValidator Contributors

UNIVERSITY OF LEICESTER

B. Variant description of family 2 by VariantValidator

VariantValidator

Submitted Variant

NM_014014.5:c.2219A>G

- Selected genome build: GRCh38
- Map location: 2q11.2
- Transcript Flag: MANE Select
- CCDS ID: CCDS2020.1

Versions

- VariantValidator 2.2.1.dev341+g0388933
- vv_hgvs 2.2.0
- VVDb vvdb_2023_8
- VVTA vvta_2023_05
- VVSeqRepo VV_SR_2023_05/master

Recommended Variant Descriptions

1. HGVS guidelines recommend using genomic and transcript descriptions in all publications
2. Use of the three- or one-letter amino acid alphabet is optional, but three-letter is recommended

Genomic descriptions

Reference Sequence Type	Variant Description
Chromosomal GRCh37	NC_000002.11:g.96957580T>C
Chromosomal GRCh38	NC_000002.12:g.96291842T>C

Transcript and protein descriptions

Reference Sequence Type	Variant Description
Transcript	NM_014014.5:c.2219A>G
Protein three letter code	NP_054733.2:p.(Asp740Gly)
Protein single letter code	NP_054733.2:p.(D740G)

Gene Information

Attribute	Identifier	Source
Symbol	SNRNP200	HGNC
Name	small nuclear ribonucleoprotein U5 subunit 200	HGNC
HGNC ID	HGNC:30859	HGNC

MANCHESTER 1824
The University of Manchester

Copyright © 2016-2023 VariantValidator Contributors

UNIVERSITY OF LEICESTER

C. Variant description of family 3 by VariantValidator

VariantValidator

Submitted Variant

NM_014014.5:c.5038C>G

- Selected genome build: GRCh38
- Map location: 2q11.2
- Transcript Flag: MANE Select
- CCDS ID: CCDS2020.1

Versions

- VariantValidator 2.2.1.dev341+g0388933
- vv_hgvs 2.2.0
- VVDb vvdb_2023_8
- VVTA vvta_2023_05
- VVSeqRepo VV_SR_2023_05/master

Recommended Variant Descriptions

1. HGVS guidelines recommend using genomic and transcript descriptions in all publications
2. Use of the three- or one-letter amino acid alphabet is optional, but three-letter is recommended

Genomic descriptions

Reference Sequence Type	Variant Description
Chromosomal GRCh37	NC_000002.11:g.96945284G>C
Chromosomal GRCh38	NC_000002.12:g.96279546G>C

Transcript and protein descriptions

Reference Sequence Type	Variant Description
Transcript	NM_014014.5:c.5038C>G
Protein three letter code	NP_054733.2:p.(Pro1680Ala)
Protein single letter code	NP_054733.2:p.(P1680A)

Gene Information

Attribute	Identifier	Source
Symbol	SNRNP200	HGNC
Name	small nuclear ribonucleoprotein U5 subunit 200	HGNC
HGNC ID	HGNC:30859	HGNC

MANCHESTER 1824
The University of Manchester

Copyright © 2016-2023 VariantValidator Contributors

UNIVERSITY OF LEICESTER

D. Variant description of family 4 by VariantValidator

VariantValidator

Submitted Variant

NM_012433.4:c.2479A>G

- Selected genome build: GRCh38
- Map location: 2q33.1
- Transcript Flag: MANE Select
- CCDS ID: CCDS33356.1

Versions

- VariantValidator 2.2.1.dev341+g0388933
- vv_hgvs 2.2.0
- VVDb vvdb_2023_8
- VVTA vvta_2023_05
- VVSeqRepo VV_SR_2023_05/master

Recommended Variant Descriptions

1. HGVS guidelines recommend using genomic and transcript descriptions in all publications
2. Use of the three- or one-letter amino acid alphabet is optional, but three-letter is recommended

Genomic descriptions

**Reference Sequence Type**	**Variant Description**
Chromosomal GRCh37	NC_000002.11:g.198266141T>C
Chromosomal GRCh38	NC_000002.12:g.197401417T>C

Transcript and protein descriptions

**Reference Sequence Type**	**Variant Description**
Transcript	NM_012433.4:c.2479A>G
Protein three letter code	NP_036565.2:p.(Arg827Gly)
Protein single letter code	NP_036565.2:p.(R827G)

Gene Information

**Attribute**	**Identifier**	**Source**
Symbol	SF3B1	HGNC
Name	splicing factor 3b subunit 1	HGNC
HGNC ID	HGNC:10768	HGNC

Copyright © 2016-2023 VariantValidator Contributors

E. Variant description of family 5 by VariantValidator

VariantValidator

Submitted Variant

NM_006842.3:c.2087C>T

- Selected genome build: GRCh38
- Map location: 11q13.1
- Transcript Flag: MANE Select
- CCDS ID: CCDS31612.1

Versions

- VariantValidator 2.2.1.dev341+g0388933
- vv_hgvs 2.2.0
- VVDb vvdb_2023_8
- VVTA vvta_2023_05
- VVSeqRepo VV_SR_2023_05/master

Recommended Variant Descriptions

1. HGVS guidelines recommend using genomic and transcript descriptions in all publications
2. Use of the three- or one-letter amino acid alphabet is optional, but three-letter is recommended

Genomic descriptions

Reference Sequence Type	Variant Description
Chromosomal GRCh37	NC_000011.9:g.65830872C>T
Chromosomal GRCh38	NC_000011.10:g.66063401C>T

Transcript and protein descriptions

Reference Sequence Type	Variant Description
Transcript	NM_006842.3:c.2087C>T
Protein three letter code	NP_006833.2:p.(Thr696Ile)
Protein single letter code	NP_006833.2:p.(T696I)

Gene Information

Attribute	Identifier	Source
Symbol	SF3B2	HGNC
Name	splicing factor 3b subunit 2	HGNC
HGNC ID	HGNC:10769	HGNC

MANCHESTER 1824 The University of Manchester

Copyright © 2016-2023 VariantValidator Contributors

UNIVERSITY OF LEICESTER

F. Variant description of family 6 by VariantValidator

VariantValidator

Submitted Variant

NM_005850.5:c.311T>C

- Selected genome build: GRCh38
- Map location: 1q21.2
- Transcript Flag: MANE Select
- CCDS ID: CCDS72900.1

Versions

- VariantValidator 2.2.1.dev341+g0388933
- vv_hgvs 2.2.0
- VVDb vvdb_2023_8
- VVTA vvta_2023_05
- VVSeqRepo VV_SR_2023_05/master

Recommended Variant Descriptions

1. HGVS guidelines recommend using genomic and transcript descriptions in all publications
2. Use of the three- or one-letter amino acid alphabet is optional, but three-letter is recommended

Genomic descriptions

Reference Sequence Type	Variant Description
Chromosomal GRCh37	NC_000001.10:g.149898663A>G
Chromosomal GRCh38	NC_000001.11:g.149926771A>G

Transcript and protein descriptions

Reference Sequence Type	Variant Description
Transcript	NM_005850.5:c.311T>C
Protein three letter code	NP_005841.1:p.(Ile104Thr)
Protein single letter code	NP_005841.1:p.(I104T)

Gene Information

Attribute	Identifier	Source
Symbol	SF3B4	HGNC
Name	splicing factor 3b subunit 4	HGNC
HGNC ID	HGNC:10771	HGNC

MANCHESTER 1824
The University of Manchester

Copyright © 2016-2023 VariantValidator Contributors

UNIVERSITY OF LEICESTER
